# A comprehensive evaluation of histopathology foundation models for ovarian cancer subtype classification

**DOI:** 10.1038/s41698-025-00799-8

**Published:** 2025-01-30

**Authors:** Jack Breen, Katie Allen, Kieran Zucker, Lucy Godson, Nicolas M. Orsi, Nishant Ravikumar

**Affiliations:** 1 Centre for Computational Imaging and Simulation Technologies in Biomedicine (CISTIB), School of Computing, University of Leeds, Leeds, UK; 2 Leeds Institute of Medical Research at St James’s, School of Medicine, University of Leeds, Leeds, UK; 3 Leeds Cancer Centre, St James’s University Hospital, Leeds, UK; 4 National Pathology Imaging Cooperative (NPIC), Leeds Teaching Hospitals NHS Trust, Leeds, UK

**Keywords:** Ovarian cancer, Cancer imaging, Mathematics and computing

## Abstract

Histopathology foundation models show great promise across many tasks, but analyses have been limited by arbitrary hyperparameters. We report the most rigorous single-task validation study to date, specifically in the context of ovarian carcinoma morphological subtyping. Attention-based multiple instance learning classifiers were compared using three ImageNet-pretrained encoders and fourteen foundation models, each trained with 1864 whole slide images and validated through hold-out testing and two external validations (the Transcanadian Study and OCEAN Challenge). The best-performing classifier used the H-optimus-0 foundation model, with balanced accuracies of 89%, 97%, and 74%, though UNI achieved similar results at a quarter of the computational cost. Hyperparameter tuning the classifiers improved performance by a median 1.9% balanced accuracy, with many improvements being statistically significant. Foundation models improve classification performance and may allow for clinical utility, with models providing a second opinion in challenging cases and potentially improving the accuracy and efficiency of diagnoses.

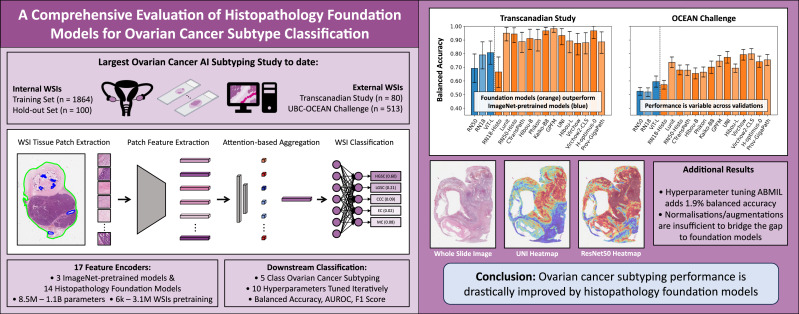

## Introduction

Ovarian cancer is the eighth most common cancer in women worldwide and typically has a poor prognosis, with 324,000 diagnosed cases translating to 207,000 deaths annually^1^. It is represented by an array of histological (morphological) subtypes with distinct prognoses and treatment options^2^. Five carcinoma subtypes account for approximately 90% of all ovarian cancers—high-grade serous (HGSC, 70%), endometrioid (EC, 11%), clear cell (CCC, 10%), low-grade serous (LGSC, 5%), and mucinous carcinomas (MC, 4%)^3–5^.

Histological subtyping is an essential component of the diagnostic process, but it can be challenging. A histopathologist will follow a systematic approach that incorporates both the macroscopic and microscopic features, which show a wide variation in ovarian carcinoma. This includes assessing the overall architecture and growth pattern of the tumour, the cytological features (for example, the shape of the cells or presence of cytoplasmic clearing), aspects important to grading like nuclear pleomorphism, presence of necrosis and mitotic activity, and additional features such as the presence of areas of metaplastic differentiation or psammoma bodies^5^. From an individual haematoxylin and eosin (H&E)-stained tissue slide, pathologists only exhibit concordance on an ovarian cancer diagnosis around 80% of the time^6^. In cases of uncertainty, a pathologist may request ancillary tests (such as immunohistochemistry) or seek a second opinion from a gynaecological subspeciality expert, which incurs associated logistical and financial burdens. With increasing cancer rates^1^ and growing complexity in diagnostic testing, histopathology services are increasingly struggling to meet demand globally. For example, most histopathology departments in the UK regularly resort to outsourcing work or hiring temporary staff^7^, despite the UK being one of the countries with the most pathologists per capita^8^. Any delays resulting from demand outstripping diagnostic resources risk catastrophic impacts on patient outcomes, with a 4-week delay in cancer treatment being associated with an approximately 10% increased mortality rate among patients^9^.

Conceptually, artificial intelligence (AI) may offer clinical value by providing a second opinion to histopathologists, streamlining the diagnostic process and offering additional support when subspecialty experts are not readily available^10^. However, AI models for ovarian cancer diagnosis have yet to demonstrate clinical utility, with most research being small-scale prototyping^11^ without regulatory approval for clinical use in Europe or the United States^12^. AI for ovarian cancer subtyping has constituted a small field of research where, aside from our work^13–15^, research has almost exclusively been published by a single group^16–23^. While the accuracy of such models has increased over time, the best models still only achieve around 80% accuracy^15,21–25^ and lack sufficient real-world testing.

One issue limiting AI in histopathology is that whole slide images (WSIs) are orders of magnitude too large for conventional (single instance) models, therefore multiple instance learning (MIL) is often employed^26^. In MIL, individual patches (the ‘instances’) are processed separately and then aggregated to learn information about a WSI. These models are impractical to train end-to-end with such large images, so frozen patch feature extractors are often used. As such, any limitation in the pretrained feature extractor can limit downstream classification performance.

In applying MIL to WSI-level classification, many researchers have used ImageNet-pretrained ResNets^27^ for patch feature extraction^13,20,28–31^. ImageNet (a set of 1.4 million natural images from 1000 classes)^32^ is popular for model pretraining as the quantity and diversity of images enable the creation of a multi-purpose feature set. However, these generic features are likely to be suboptimal and computationally inefficient when applied to histopathology images, which contain a relatively homogeneous and restricted set of shapes and colours, with subtle differences being relevant to diagnostic decisions^5,33^.

Recently, many researchers have attempted to create histopathology ‘foundation models’, using self-supervised learning (SSL) techniques to generate broad histopathological feature sets which are not specific to a single organ/cancer type. These approaches have grown rapidly, from tens of thousands of WSIs used to train models with tens of millions of parameters in 2022 and early 2023^34–39^ to millions of WSIs^40–42^ and billions of parameters more recently^25,43^. Foundation models have typically been based on vision transformers (ViTs), utilising the impressive scalability of transformers seen across many fields, most notably with large language models^44,45^. Histopathology foundation models have exhibited impressive performance across diverse tasks^37,46–49^ including ovarian cancer subtyping^15,24,25^, although analyses have been relatively shallow, without thorough hyperparameter tuning and rigorous statistical comparison of downstream models. Consequently, it is unclear whether models were applied optimally (especially those exhibiting suboptimal performance), and whether the differences between them were significant. Furthermore, many analyses have been conducted using single-centre data, limiting the assessment of models’ generalisability.

In this study, we present the most comprehensive validation conducted to date comparing feature extraction methods for ovarian cancer subtyping, including three ImageNet-pretrained feature extractors and fourteen histopathology foundation models. The analysis includes rigorous hyperparameter tuning and evaluations through five-fold cross-validation, hold-out testing, and external validations, and was conducted with the largest collection of ovarian cancer WSIs used in any AI validation study to date. We further investigate whether the classification performance of the ImageNet-pretrained ResNet50 features can match those of the foundation models through stain normalisation, tissue augmentation, or different tissue detection techniques.

## Methods

### Ovarian carcinoma histopathology data

A training set of 1864 formalin-fixed, paraffin-embedded (FFPE) adnexal tissue WSIs was retrospectively collected from 434 cases of ovarian carcinoma treated at Leeds Teaching Hospitals NHS Trust between 2008 and 2022. Cases were only included if a gynaecological pathologist had diagnosed them as one of the five most common epithelial ovarian cancer subtypes (HGSC, LGSC, CCC, MC, EC). A histopathologist (K.A.) independently verified all diagnoses, removing any cases with discrepancies. Several representative H&E-stained adnexal tissue glass slides were selected for each case, cleaned, anonymised, and digitised at 40× magnification using a Leica Aperio AT2 scanner. The population-level class imbalance was reflected in the training set (Table 1), with the least common subtype (LGSC) represented by only 92 WSIs from 21 cases, compared to 1266 WSIs from 308 cases for the most common subtype (HGSC).

**Table 1:** Composition of training and validation datasets

Carcinoma subtype	Training WSIs (patients)	Hold-out WSIs (patients)	Transcanadian^33^ WSIs (patients)	OCEAN^50^ WSIs
High-grade serous (HGSC)	1266 - 68% (308)	20 - 20% (7)	30 - 38% (30)	217 - 42%
Low-grade serous (LGSC)	92 - 5% (21)	20 - 20% (6)	9 - 11% (9)	42 - 8%
Clear cell (CCC)	198 - 11% (45)	20 - 20% (7)	20 - 25% (20)	94 - 18%
Endometrioid (EC)	209 - 11% (38)	20 - 20% (5)	11 - 14% (11)	119 - 23%
Mucinous (MC)	99 - 5% (22)	20 - 20% (5)	10 - 13% (10)	41 - 8%
Total	1864 (434)	100 (30)	80 (80)	513

The breakdown of each carcinoma subtype in the training (cross-validation) set, independent internal hold-out test set, and external validation sets. Numbers in brackets indicate the number of unique patients where this is known. Percentages indicate the relative proportions of WSIs.

An independent class-balanced hold-out test set was collected through the same protocol, consisting of 100 primary surgery specimen WSIs from 30 patients. Two additional external test sets were also used. The *Transcanadian Study* dataset^33^ consisted of 80 WSIs from 80 patients, which had been digitised using an AperioScope scanner and made available at 20× magnification alongside subtype labels that had been determined by a gynaecological pathologist. The *OCEAN Challenge* dataset contained 513 WSIs that had been labelled as one of the five main ovarian carcinoma subtypes. This was a highly heterogeneous dataset, with tissue prepared and digitised across many different labs. However, information was not provided concerning how ground-truth labels were determined and which tissue types were included.

The main aim of this study was to classify the subtype of primary surgery specimens, which typically have the highest diagnostic quality. Interval debulking surgery (IDS) samples may be impacted by prior treatments (particularly chemotherapy) and are thus presumed to have less reliable morphological features. The internal hold-out and Transcanadian Study validation sets contained only primary surgery specimens. It was unclear which samples were included in the OCEAN challenge dataset, though this set was intentionally diverse^50^, so likely included both primary surgery and IDS samples. The training set contained both primary (1412 WSIs) and IDS specimens (452 WSIs), as we have previously found the latter to be beneficial in supplementing training data^51^.

### Slide classification pipeline

**Fig. 1:**
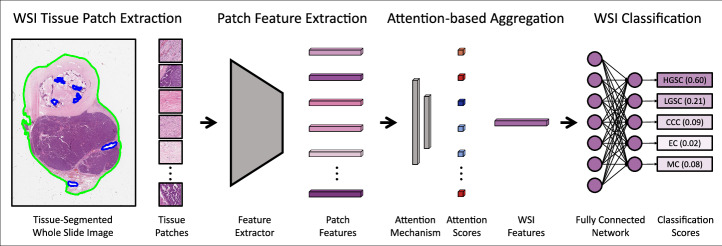
Classification model pipeline. Attention-based multiple instance learning (ABMIL) classifier for ovarian cancer subtyping, showing the classification of a high-grade serous carcinoma (HGSC).

Slide classification was performed using an attention-based multiple instance learning (ABMIL)^52^ classification pipeline (Fig. 1), one of the most commonly used slide classification techniques in contemporary research^53^. WSI preprocessing and patch extractions were performed using the CLAM default procedures^28^. First, tissue was segmented from plain background using saturation thresholding, where only the pixels with saturation higher than the threshold (8/255) were labelled as tissue. Then, non-overlapping 1024 × 1024 pixel tissue patches were extracted at the native 40× tissue magnification and downsampled to 256 × 256 pixels at 10× apparent magnification, which we previously found to be optimal for this task when using the ResNet50 encoder^14^. For external data, 512 × 512 pixel tissue patches were extracted at the native 20× magnification and downsampled to achieve the same 256 × 256 pixels at 10× apparent magnification. Features were then extracted from these patches following the specific procedure of each feature extraction model, which typically involved first applying a standard normalisation to the red-green-blue (RGB) colour channels, and for ViT-based models typically also involved resizing or cropping patches to 224 × 224 pixels.

Patch features were then used to train an ABMIL classifier for each feature extractor. In ABMIL, the patch features were passed through a trainable attention layer which assigned each patch an attention score (between 0 and 1) representing the relative importance of the patch in downstream classification. An attention-weighted average of the patch features generated WSI-level features, which were classified through a fully connected neural network with one output node per class. The outputs were passed through the softmax function to generate the (uncalibrated) classification probabilities for each subtype, with the maximum taken as the predicted class.

### Feature extraction models

A total of seventeen patch feature extractors were compared (Table 2), three of which had been trained through the traditional approach of supervised classification on ImageNet data^32^, and the other fourteen had been trained using histopathology images through various self-supervised learning (SSL) approaches. All feature extractors were available online, with some requiring approval before they could be accessed.

**Table 2:** Summary of the seventeen feature extraction models

Feature extractor	Backbone	Data type	Data source	Pretraining algorithm	Pretraining images	Pretraining magnification(s)	Parameters	Patch features
RN50^27^	ResNet50	Natural	ImageNet-1k	Supervised	1,431,167	NA	8,543,296	1024
RN18^27^	ResNet18	Natural	ImageNet-1k	Supervised	1,431,167	NA	11,176,512	512
ViT-L^54^	ViT-L	Natural	ImageNet-21k	Supervised	14,197,122	NA	303,301,632	1024
RN18-Histo^34^	ResNet18	Histology	57 Online Sets	SimCLR	>25,000 WSIs	10×, 20×, 40×, 100×	11,176,512	512
Lunit^37^	ViT-S	Histology	TCGA + Internal	DINO	36,666 WSIs	20×, 40×	21,670,272	384
RN50-Histo^37^	ResNet50	Histology	TCGA + Internal	Barlow Twins	36,666 WSIs	20×, 40×	23,508,032	2048
CTransPath^36^	CNN + SwinT	Histology	TCGA + PAIP	Novel SSL	32,220 WSIs	20×	27,520,038	768
Hibou-B^40^	ViT-B	Histology	Internal	DINOv2	1,141,581 WSIs	Unclear	85,741,056	768
Phikon^38^	ViT-B	Histology	TCGA	iBOT	6093 WSIs	20×	85,798,656	768
Kaiko-B8^55^	ViT-B	Histology	TCGA	DINO	~29,000 WSIs	5×, 10×, 20×, 40×	85,807,872	768
GPFM^24^	ViT-L	Histology	47 Online Sets	Novel Distillation	72,280 WSIs	Unclear	303,228,928	1024
UNI^47^	ViT-L	Histology	Internal + GTEx	DINOv2	100,426 WSIs	20×	303,350,784	1024
Hibou-L^40^	ViT-L	Histology	Internal	DINOv2	1,141,581 WSIs	Unclear	303,659,264	1024
Virchow^41^	ViT-H	Histology	Internal	DINOv2	1,488,550 WSIs	20×	631,229,184	2560
Virchow2-CLS^42^	ViT-H	Histology	Internal	DINOv2	3,134,922 WSIs	5×, 10×, 20×, 40×	631,239,424	1280
H-optimus-0^43^	ViT-g	Histology	Internal	DINOv2	>500,000 WSIs	20×	1,134,774,272	1536
Prov-GigaPath^25^	ViT-g	Histology	Internal	DINOv2	171,189 WSIs	20×	1,134,953,984	1536

There are three ImageNet-pretrained models and fourteen histopathology foundation models. These are grouped by data type and ordered by increasing model size.

The ImageNet-pretrained models were a ResNet50 (RN50)^27^, ResNet18 (RN18)^27^, and a large vision transformer (ViT-L)^54^. The ResNet50 outputs were taken from the end of the third residual block (as in CLAM^28^) to give 1024 features per input patch. The ResNet18 does not have a layer this large, so 512 features were extracted from the end of the fourth residual block instead. ViT-L was applied without a final fully connected layer to give 1024 features per patch. ImageNet-pretraining for ResNet models had been conducted using the original 1000 class ImageNet dataset alone, whereas the ViT-L was first trained on the much larger set of nearly 22,000 classes, and then fine-tuned to the same set of 1000 classes. The reported ImageNet classification accuracies were 80.9%, 69.8%, and 85.1% for ResNet50, ResNet18, and ViT-L, respectively.

The SSL pretraining of the foundation models allowed large quantities of diverse data to be leveraged without the need for extensive labelling. One of the earliest histopathology foundation models was a ResNet18 trained through a self-supervised strategy with 57 online datasets in 2021^34^, which we refer to as ‘RN18-Histo’. A similar approach was taken in a subsequent study to pre-train a ResNet50 with a combination of TCGA and proprietary data, which we refer to as ‘RN50-Histo’^37^. Another early approach, CTransPath^36^, used a novel backbone which combined a CNN with a Swin Transformer and pretrained these through a novel SSL strategy using multiple online datasets.

Newer histopathology foundation models have typically used vision transformer backbones. The smallest such model, Lunit^37^, was based on the small vision transformer backbone (ViT-S), which gave a model of a similar size as RN50-Histo that had been pretrained on the same dataset (using DINO). Three of the foundation models were built using the base vision transformer (ViT-B) backbone with different pretraining procedures, with Phikon^38^ trained using iBOT on a small subset of TCGA data, Kaiko-B8^55^ on a much larger set of TCGA data using DINO, and Hibou-B^40^ on a huge proprietary dataset using DINOv2. The authors of Kaiko-B8 also made their model available with four other backbone sizes, though the B8 variation gave the best overall performance in their evaluations^55^. Hibou-B was included as it was the best-available version of this model when initial validations were conducted, although the authors reported their larger model, Hibou-L, to have given better performance^40^.

The largest histopathology foundation models (all published in 2024) have typically trained larger vision transformers with proprietary datasets of over 50,000 WSIs using DINOv2^56^. GPFM^24^, UNI^47^, and Hibou-L are large vision transformers (ViT-L) trained with 72,280 WSIs, 100,426 WSIs, and 1,141,581 WSIs, respectively. Virchow^41^ and its recent update, Virchow2^42^, are huge vision transformers (ViT-H) trained with the largest dataset for any histopathology foundation model to date, with nearly 1.5m WSIs in the first version and over 3m WSIs in the second version. Virchow also has the largest feature space as the class tokens are concatenated with the average patch tokens, where typically only the class tokens would be used. As Virchow2 was reported by the original authors to give better results using just the class tokens^42^, we adopted this version as ‘Virchow2-CLS’.

Prov-GigaPath^25^ and H-optimus-0^43^ were the largest accessible histopathology foundation models by far, with the ViT-g backbone giving over one billion parameters, nearly twice as many as the next largest (Virchow2-CLS), and over 100× as many parameters as the smallest foundation model (RN18-Histo). These models had also been trained with hundreds of thousands of WSIs. Prov-GigaPath includes a patch-to-slide aggregator, though we focused only on the patch feature extractor.

### Normalisation and augmentation analysis

Previous studies have often used normalisations and augmentations to attempt to improve the robustness of models based on ImageNet-pretrained CNNs^57^, including models for ovarian cancer subtyping^20,21^. To investigate whether the baseline ImageNet-pretrained ResNet50 encoder could be made competitive with the modern alternatives, we applied this feature extractor with a variety of data preprocessing techniques. Seven approaches were evaluated, with two applying stain normalisations (Reinhard^58^ and Macenko^59^), two applying Otsu thresholding^60^ for adaptive tissue detection (with and without Macenko normalisation), and three applying colour augmentations (increasing the apparent dataset size by factors of 5×, 10×, and 20×). Examples of these procedures are presented in Supplementary Figs. 2–4.

### Hyperparameter tuning and evaluation procedures

ABMIL classifiers were tuned using an iterative grid search where typically two hyperparameters were adjusted at a time, with the best taken forward to the next iteration. Ten total hyperparameters were tuned using the average loss of the five-fold validation sets. Seven of these hyperparameters directly influenced the Adam optimiser^61^, controlling the learning rate, learning rate decay proportion and patience, first and second moment decay, optimisation stability, and L2 regularisation rate. The remaining hyperparameters controlled the model size (the dimension of the attention layer and subsequent fully connected layer), and the proportions of parameter dropout and data dropout during training. Models were trained using a balanced cross-entropy loss and class-weighted sampling to help account for the class imbalance in the training set. Initial hyperparameters were determined based on our previous study in which ABMIL was tuned using ResNet50 features for the same task with a smaller dataset^14^. Over 150 unique hyperparameter configurations were evaluated during the tuning of each classifier.

Models were evaluated using the balanced accuracy, macro-averaged area under the receiver operating characteristic curve (AUROC), and macro F1 score. These metrics assessed different aspects of classification performance, with AUROC giving a holistic but imbalanced overview of discriminative power, F1 giving a balanced measure of predictive performance at a specific threshold, and balanced accuracy representing realistic clinical performance. Stratified five-fold cross-validation (split 60-20-20 train-val-test at the case level to avoid data leakage) was employed during training. In hold-out testing and external validations, the predictions of the five cross-validation models were averaged to generate an ensembled classification. All results were reported using the mean and 95% confidence intervals from 10,000 iterations of bootstrapping.

Model efficiency was evaluated as the average time to preprocess and classify a WSI using a consistent class-balanced set of 20 WSIs from the internal hold-out test set, with the evaluation repeated three times for each model and the median result used to account for variability. An ablation study was also conducted to investigate whether hyperparameter tuning improved model performance, with the performance of the tuned models compared to those using the default hyperparameters.

Paired *t*-tests were used to test for statistically significant differences between each model and the baseline ResNet50 across the five cross-validation folds (before bootstrapping), with *p*-values adjusted for multiple testing using a false discovery rate correction^62^. Results were considered *statistically significant* given an adjusted *p*-value < 0.05. The same approach was used to compare whether differences in the results between each pair of foundation models were significant. Paired *t*-tests were also used in the hyperparameter tuning ablation to determine whether tuning the ABMIL classifiers had a statistically significant effect on the final results.

This manuscript was prepared following the TRIPOD+AI (Transparent Reporting of a multivariable prediction model for Individual Prognosis Or Diagnosis + Artificial Intelligence) checklist^63^ to ensure thorough reporting, with the completed checklist available in Supplementary Figs. 7, 8. The PyTorch-based code used in this study is available at https://github.com/scjjb/Ovarian_Features. Experiments were conducted using an NVIDIA A100 GPU and 32 AMD EPYC7742 CPUs @3.4GHz.

## Results

### Foundation model performance

**Fig. 2:**
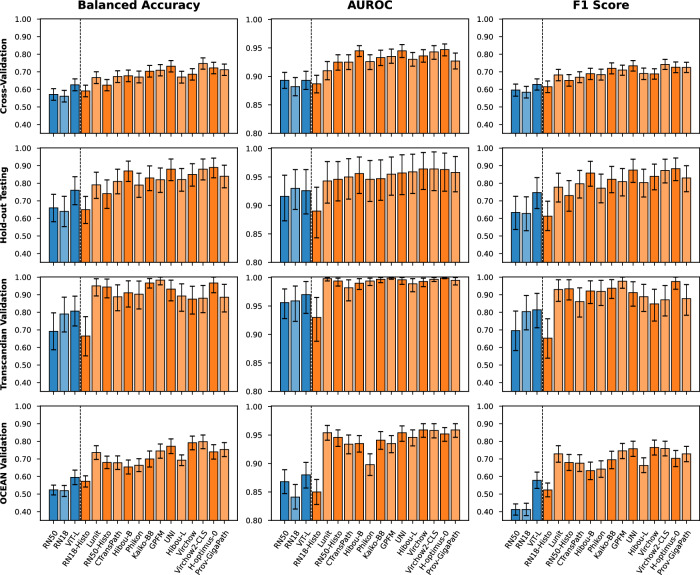
Ovarian cancer subtyping results. The mean and 95% confidence interval generated by 10,000 iterations of bootstrapping for each metric. Blue indicates ImageNet-pretrained feature extractors and orange indicates histopathology foundation models. Hold-out testing and external validation results are based on an ensemble of cross-validation models. Precise values are provided in Supplementary Tables 3–6.

As shown in Fig. 2, no single model gave the greatest results in every validation, with Virchow2-CLS giving the greatest performance in cross-validation, H-optimus-0 in hold-out testing and the Transcanadian Study external validation, and Virchow in the OCEAN Challenge external validation. RN18-Histo had the worst performance of any foundation model in all validations and was the only foundation model to perform worse than an ImageNet-pretrained encoder overall.

**Fig. 3:**
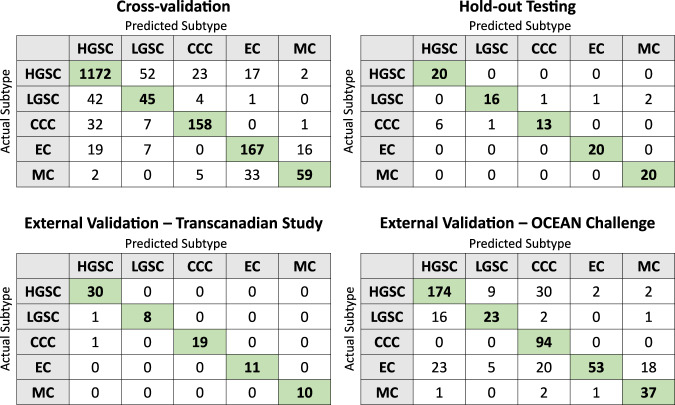
Optimal confusion matrices. The confusion matrix from each validation for the optimal ABMIL classifier with features from the H-optimus-0 foundation model. Correct classifications are indicated in green.

The H-optimus-0 model achieved the greatest averaged performance across all validations (Table 3), with 83.0% average balanced accuracy, 0.965 average AUROC, and 0.822 average F1 score. This performance was closely followed by that of UNI and Virchow2-CLS. The worst averaged performances were given by CNN-based feature extraction models (RN50, RN18, RN18-Histo), followed by the ImageNet-pretrained vision transformer. Confusion matrices for the optimal H-optimus-0 model (Fig. 3) show that no single class was the best (or worst) classified across all validations. The worst F1 scores were found for the classification of LGSC in cross-validation (0.443) and the OCEAN Challenge validation (0.582) and for EC in the OCEAN Challenge validation (0.606). In these validations, LGSC was often confused with HGSC, and there was a moderate level of confusion between EC and MC. Further class-level results are provided in Supplementary Table 7.

**Table 3:** Averaged results across all four validations

	Feature extractor	Balanced accuracy	AUROC	F1 score	Avg inference time (s/WSI)
ImageNet-pretrained models	RN50	61.2%	0.908	0.585	75.6
	RN18	62.8%	0.903	0.607	75.4
	ViT-L	69.7%	0.917	0.692	99.3
Histopathology foundation models	RN18-Histo	62.0%	0.889	0.601	76.1
	Lunit	78.6%	0.951	0.780	76.4
	RN50-Histo	74.7%	0.953	0.749	75.1
	CTransPath	76.2%	0.948	0.751	75.7
	Hibou-B	77.9%	0.957	0.775	76.9
	Phikon	75.7%	0.941	0.754	76.9
	Kaiko-B8	80.0%	0.955	0.794	129.0
	GPFM	81.4%	0.956	0.811	125.1
	UNI	82.9%	0.963	0.820	99.9
	Hibou-L	76.9%	0.956	0.762	130.4
	Virchow	80.1%	0.963	0.785	243.1
	Virchow2-CLS	82.6%	**0.966**	0.811	245.8
	H-optimus-0	**83.0%**	0.965	**0.822**	425.0
	Prov-GigaPath	79.8%	0.960	0.791	319.8

The average inference times were measured on a fixed subset of the internal hold-out test set. The greatest result for each metric is shown in bold.

The difference in performance between each foundation model (except RN18-Histo) and the baseline ImageNet-pretrained ResNet50 was found to be significant by all metrics in all validations, except by the AUROC in cross-validation (for nine foundation models), RN50-Histo by most metrics in internal validations, and Hibou-B by balanced accuracy in the OCEAN external validation. There was no significant difference between the performance of the baseline model and either the RN18 feature extractor or the RN18-Histo foundation model in most validations. The difference between the baseline ResNet50 and the ViT-L feature extractor was statistically significant in most validations for the balanced accuracy and F1 score, but not the AUROC. The *p*-values are tabulated in Supplementary Table 16.

The difference in performance between each pair of foundation models was typically not significant after adjusting for multiple testing. There were no significantly different models for all three metrics in cross-validation, though there were 27 such significant pairs of models in hold-out testing, 1 in the external validation on the Transcanadian Study dataset, and 10 in the external validation on the OCEAN Challenge dataset, with 29 of the 91 total pairs of models being significantly different for all three metrics in at least one validation. The models which most frequently had significantly worse performance than other models were RN18-Histo (worse than all other models except RN50-Histo and Phikon) and Phikon (worse than six other models), and the models which most frequently significantly outperformed other models were Virchow2-CLS (better than six other models) and H-optimus-0 (better than five other models). All significant results are presented in Supplementary Table 18.

**Fig. 4:**
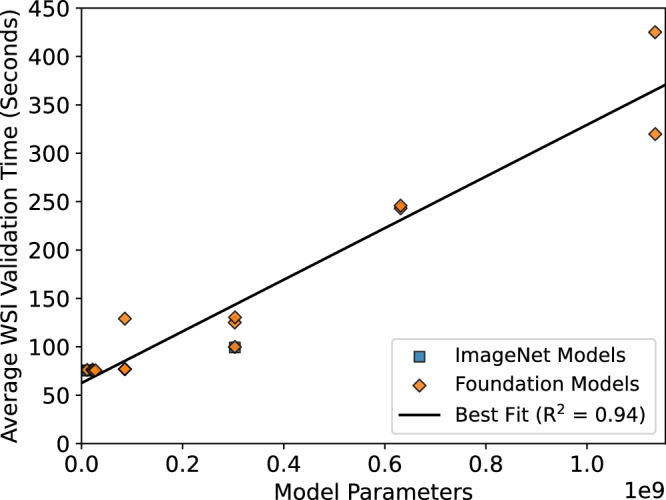
Model inference times. The average inference time per WSI for each model, including tissue patch extraction, feature encoding, and ABMIL classification time.

There was a strong positive relationship (*R*^2^ = 0.93) between the size of feature extraction models and the computational runtime (Fig. 4). The most computationally efficient models were typically the smallest, with an average inference time per WSI between 75 and 77 seconds for each of the ResNets, Lunit, CTransPath, Hibou-B, and Phikon models (Table 3). Feature encoding was the slowest step of slide inference, taking over 90% of the total computational time for all models, with the remaining time divided between the initial tissue patch extraction and the subsequent forward pass of patch features through the trained ABMIL classifiers. The average inference times did not vary greatly for any model over the three repeats, with a maximum range of 1.7s (75.3–77.0s) per WSI from the CTransPath model. The largest models were the slowest overall, with Prov-GigaPath averaging 320s and H-optimus-0 averaging 425s per WSI, over 5 times as long as the fastest models. These largest feature extractors also required much greater computational resources (particularly VRAM) as they were each over 4GB in size, whereas the smallest models were each under 100MB (RN50, RN18, RN18-Histo, Lunit, RN50-Histo).

### Normalisation and augmentation results

**Fig. 5:**
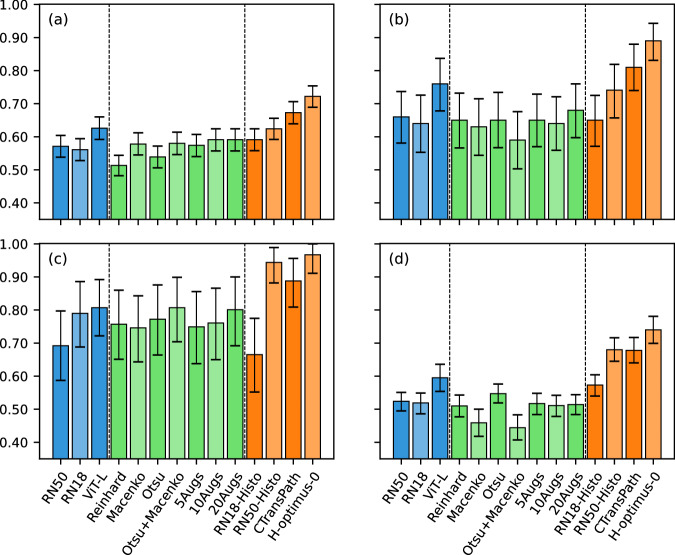
Preprocessing analysis results. Comparison of the balanced accuracy for each ImageNet-pretrained feature extractor (blue), the seven ResNet50 models with varied preprocessing techniques (green), and the three worst-performing (RN18-Histo, RN50-Histo, and CTransPath) and the single best-performing foundation models (H-optimus-0) in (**a**) cross-validation, (**b**) hold-out testing, (**c**) external validation on the Transcanadian Study dataset, (**d**) external validation on the OCEAN Challenge dataset. For validations (**b**)–(**d**), predictions were ensembled from the five cross-validation models. Results reported as the mean and 95% confidence interval generated by 10,000 iterations of bootstrapping. Precise values and other metrics are presented in Supplementary Tables 12–15.

As shown in Fig. 5, different preprocessing techniques had inconsistent effects on the baseline ResNet50 feature extractor, with modest benefits in internal validations, and variable effects in external validations. In cross-validation, no preprocessing method improved the balanced accuracy or F1 score by more than 0.02, and no improvement was seen in AUROC with any method. In hold-out testing, only the 20× colour augmentation improved performance, increasing F1 by 0.023 and balanced accuracy by 0.020, but reducing AUROC by 0.012. However, in the external validation on the Transcanadian Study dataset, every preprocessing method improved performance compared to the baseline by over 0.05 balanced accuracy and F1 score and over 0.002 AUROC. The greatest performances in this validation were found by combining Otsu thresholding with Macenko normalisation and by 20× colour augmentations, which each increased the F1 score and balanced accuracy above baseline performance by over 0.1, and AUROC by over 0.016. For the OCEAN Challenge external validation, most preprocessing methods gave worse results than the baseline approach, with only Otsu thresholding providing any benefit over the baseline performance.

Despite some modest improvements offered by different preprocessing techniques, particularly in the Transcanadian Study external validation, the best-performing model based on the ImageNet-pretrained ResNet50 backbone was still outperformed by every foundation model (except RN18-Histo) in every validation. Furthermore, none of the different preprocessing methods gave statistically significant differences in performance compared to the baseline approach in any validation. The full results are tabulated in Supplementary Tables 12–15.

### Hyperparameter tuning ablation results

**Fig. 6:**
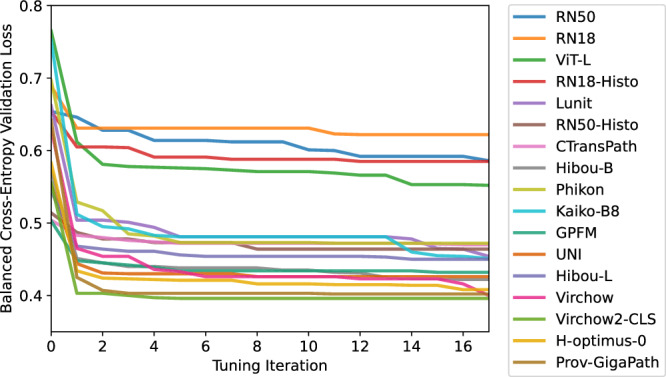
Validation losses. The average validation loss from five-fold cross-validation for each model across each hyperparameter tuning iteration.

Hyperparameter tuning improved the average validation loss for every model by at least 0.034 (CTransPath from 0.504 to 0.470), with a median improvement of 0.150, and a maximum of 0.301 (Kaiko-B8 from 0.752 to 0.451). As shown in Fig. 6, the majority of this benefit was found within the first iteration of hyperparameter tuning for every model (except the ImageNet-pretrained ResNet50), with a median improvement of 0.121 validation loss from tuning only the learning rate and ABMIL model size. The hyperparameters adjusted in tuning and used in the final models are provided in Supplementary Tables 1 and 2.

**Fig. 7:**
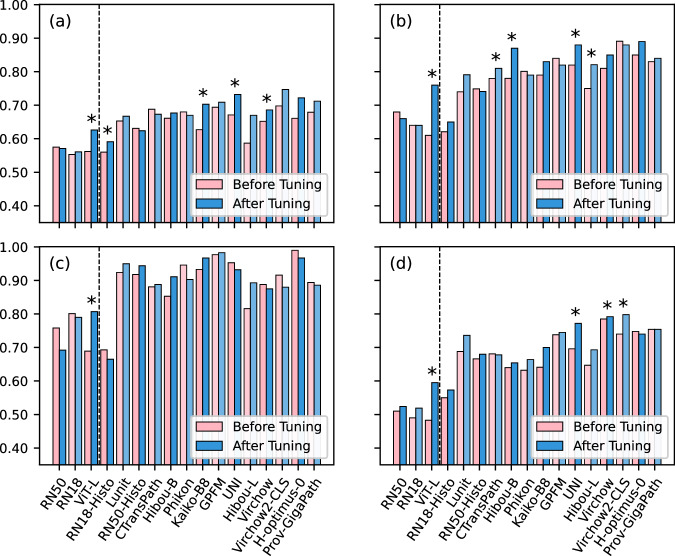
Results of hyperparameter tuning. The balanced accuracy compared for each ABMIL model trained with the default hyperparameters (pink) and the tuned hyperparameters (blue) in (**a**) cross-validation, (**b**) hold-out testing, (**c**) external validation on the Transcanadian Study dataset, and (**d**) external validation on the OCEAN Challenge dataset. For validations (**b**)–(**d**), predictions were ensembled from the five cross-validation models. *Indicates a significant difference in the paired *t*-test at the 5% significance level.

The balanced accuracy of the tuned ABMIL classifiers is compared to the untuned models (using default hyperparameters) in Fig. 7, with exact values and other metrics provided in Supplementary Tables 8–11. The median impact of hyperparameter tuning was an improvement of 1.9% balanced accuracy, 0.005 AUROC, and 0.025 F1 score, though the effect on any given model was variable, with balanced accuracies changed by −6.6% to +15.0%, AUROCs by −0.013 to +0.041, and F1 scores by −0.073 to +0.146. The only models which did not benefit from hyperparameter tuning were those using the ResNet50, ResNet18, Phikon, and H-optimus-0 feature extractors. All other models had a statistically significant difference between tuned and untuned results in at least one evaluation (Supplementary Table 17), with these significant differences only occurring in cases where tuning improved performance. The extent of the benefits varied across validations, with a median change in balanced accuracy of +3.1% in cross-validation, +3.0% in hold-out testing, −0.8% in the Transcanadian Study external validation, and +1.9% in the OCEAN Challenge external validation. The only models to significantly benefit in every validation were the ImageNet-pretrained ViT-L and Hibou-L, though these benefits were not present for every metric.

## Discussion

In this study, we thoroughly compared the effects of different patch feature extractors on the slide-level classification of ovarian carcinoma morphological subtypes. The results indicated that transformer-based histopathology foundation models improved downstream classification when compared to non-domain-specific and ResNet-based feature extractors, with 13 out of 14 foundation models outperforming all ImageNet-pretrained models in all evaluations. The only foundation model which did not exceed ImageNet-pretrained model performance was RN18-Histo, which was the single worst-performing model in hold-out testing and the Transcanadian Study external validation, though it did outperform the ImageNet-pretrained ResNet models in the other two validations. RN18-Histo was the earliest published histopathology foundation model, and as such, it was one of the few foundation models to not use a transformer-based backbone. In this study, RN18-Histo was also the smallest foundation model, had the second-smallest feature space, and was pretrained with the second-smallest dataset.

**Fig. 8:**
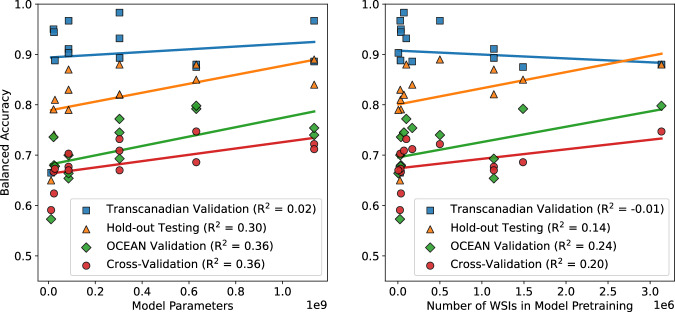
Accuracy compared to efficiency. Balanced accuracy results for each histopathology foundation model-based classifier in each validation shown in relation to the number of model parameters and number of WSIs used in the pretraining of the foundation model. The line of best fit and the corresponding coefficient of determination (*R*^2^) are provided for each validation.

As shown in Fig. 8, in most validations, there was a slight positive relationship between performance (specifically, balanced accuracy) and each of the foundation model size and pretraining dataset size. These relationships were fairly weak, with the relationship between performance and foundation model size having *R*^2^ values between 0.02 and 0.36, and the relationship between performance and pretraining dataset size between −0.01 and 0.24. The greatest performance in most validations was achieved by one of the largest models (Virchow, Virchow2-CLS, H-optimus-0), though in the Transcanadian Study external validation the smaller GPFM model performed best, and the single largest model (Prov-GigaPath) did not achieve optimal results in any validation. Three models were trained with over one million WSIs, with two being among the best-performing models (Virchow, Virchow2-CLS), and the other being one of the worst-performing ViT-based foundation models overall (Hibou-B).

To investigate which foundation models outperformed expectations, we investigated which models had positive residuals of at least 1% when compared to the lines of best fit in Fig. 8. UNI and Kaiko-B8 consistently performed better than expected given their foundation model size, with GPFM and Virchow2-CLS also performing better than expected in three of the four validations. The UNI and GPFM models consistently performed better than expected given the pretraining dataset size, with Kaiko-B8, Virchow2-CLS and H-optimus-0 all better than expected in three of the four validations. These results indicate that UNI is particularly data-efficient and computationally efficient for a foundation model of its ability. Where the H-optimus-0 classifier took an average of 425s per WSI, UNI took only 100s (24% as long) with a reduction of only 0.1% average balanced accuracy across the four validations (Table 3). It was not clear how UNI outperformed expectations in this way, with similar overall methodologies employed in training models which did not achieve such great results. The proportion of gynaecological WSIs in the UNI training set (5.8%) was exceeded in the training of several other models^24,34,38,40,41^, though for most models it was not clear what proportion of the training set was specifically composed of the five subtypes of interest, so it was not clear whether this was an influential factor.

Different preprocessing techniques often had little impact on internal performance (likely due to the homogeneity of the single-centre dataset) and on the OCEAN Challenge validation, but they did aid the generalisability to the Transcanadian Study dataset. There was a modest positive trend between the number of augmentations used and the resulting model performance which may continue beyond the 20× augmentations used herein, though this may not be worth the considerable associated computational burden since the normalisation approaches achieved a similar level of performance. No individual normalisation, augmentation, or tissue detection approach consistently improved performance, with each giving worse performance than the baseline in at least one validation. As such, we believe there is much greater value in selecting the optimal feature extractor than there is in applying varied preprocessing techniques in the training of a downstream classifier. This conclusion was also found in a recent study^64^ which investigated 14 different feature extractors using ABMIL in the context of breast and colorectal cancers (without hyperparameter tuning).

Hyperparameter tuning the downstream ABMIL classifier had a modest but often significantly beneficial effect on classification performance. The variability in the benefits may reflect both the fitness of the originally selected hyperparameters and the versatility of the models. The original hyperparameters were taken from our previous study using the ImageNet-pretrained ResNet50 feature extractor for the same task^14^, so the hyperparameters were likely better suited to this feature extractor than those which used different architectures and training datasets. Most of the benefit of hyperparameter tuning on the validation loss was achieved by adjusting the learning rate and the size of the ABMIL classifier, so just tuning these may be a more computationally efficient approach to improve model performance and the robustness of validations.

Classification performance was generally higher in hold-out testing than in cross-validation and was higher still in the Transcanadian Study external validation. However, the OCEAN external validation gave similar performance to that of cross-validation. This may be influenced by the diagnostic quality of the data, with the internal cross-validation dataset incorporating post-chemotherapy WSIs and the OCEAN dataset being unclear in this regard. Validations using only staging data achieved optimal balanced accuracies of 89% and 97%, compared to only 75% and 80% in the validations potentially including IDS samples (which can feature chemotherapy-induced morphological changes, such as varying amounts of cell death and associated changes in surrounding stroma). In cross-validation, the H-optimus-0 balanced accuracy for IDS samples was only 64.7% (with all EC slides incorrectly classified), compared to 71.0% for primary surgery samples (Supplementary Fig. 1). The challenge posed by neoadjuvant treatment is recognised by pathologists, and it is recommended in these cases that tumour subtyping is performed using pre-treatment biopsies rather than resection specimens^65^.

Two pathologists (K.A. and N.M.O.) reviewed a subset of 100 WSIs in the OCEAN set and found that eight exhibited extensive tissue microarray coring, two were almost entirely necrotic, and one displayed image stitching problems. Furthermore, the staining and colour balance were inconsistent across this cohort, which comprised both biopsies and resection specimens. These characteristics may have contributed to the poorer performance noted on the OCEAN dataset. In contrast, the Transcanadian Study set contained a single representative staging slide of the tumour per patient, and the slides were largely devoid of artefacts. This particularly high-quality data may represent a best-case research scenario, rather than a more realistic representation of the variable quality and tumour content of clinical slides, where guidance recommends the sampling of heterogeneous areas of tumour that have the potential to compromise the quality of slide preparation and interpretation, such as calcification or necrosis. The hold-out and external validations likely also benefitted from the five-fold ensembled predictions when compared to the five-fold cross-validation. While this is the most comprehensive study of AI ovarian cancer subtyping to date, the relatively small size of the test sets still results in a high level of uncertainty, as reflected by the wide confidence intervals. Thus, part of the difference in performance between datasets may be attributed to random chance.

The results of this study are similar to those of the only previous studies to use large ovarian cancer subtyping datasets (each with around 1000 WSIs)^21–23^. One study presented a multi-scale graph model^22^ and reported an optimal cross-validation balanced accuracy of 73% and F1 score of 0.69, respectively. Another^21^ evaluated four MIL approaches and reported an optimal cross-validation balanced accuracy of 81%, AUROC of 0.95, and F1 score of 0.79. In an external validation using an ensemble of cross-validation models on 60 WSIs, the authors reported a balanced accuracy of 80%, AUROC of 0.96, and F1 score of 0.81. The final study focused on adversarial domain adaptation^23^ and achieved optimal internal and external balanced accuracies of 80% and 83% from a CTransPath-based MIL classifier. Other studies applying foundation models to ovarian cancer subtyping have reported optimal balanced accuracies of 82% and ~88% using UNI on the OCEAN dataset and Prov-GigaPath on an internal dataset, respectively^24,25^. These comparisons are provided for context and should not be considered to be conclusive given the differences in the datasets used. A sparsity of publicly available data has limited external validations in most previous research^11^, and for the largest accessible dataset (the OCEAN Challenge set) very little information has been provided about the data provenance.

**Fig. 9:**
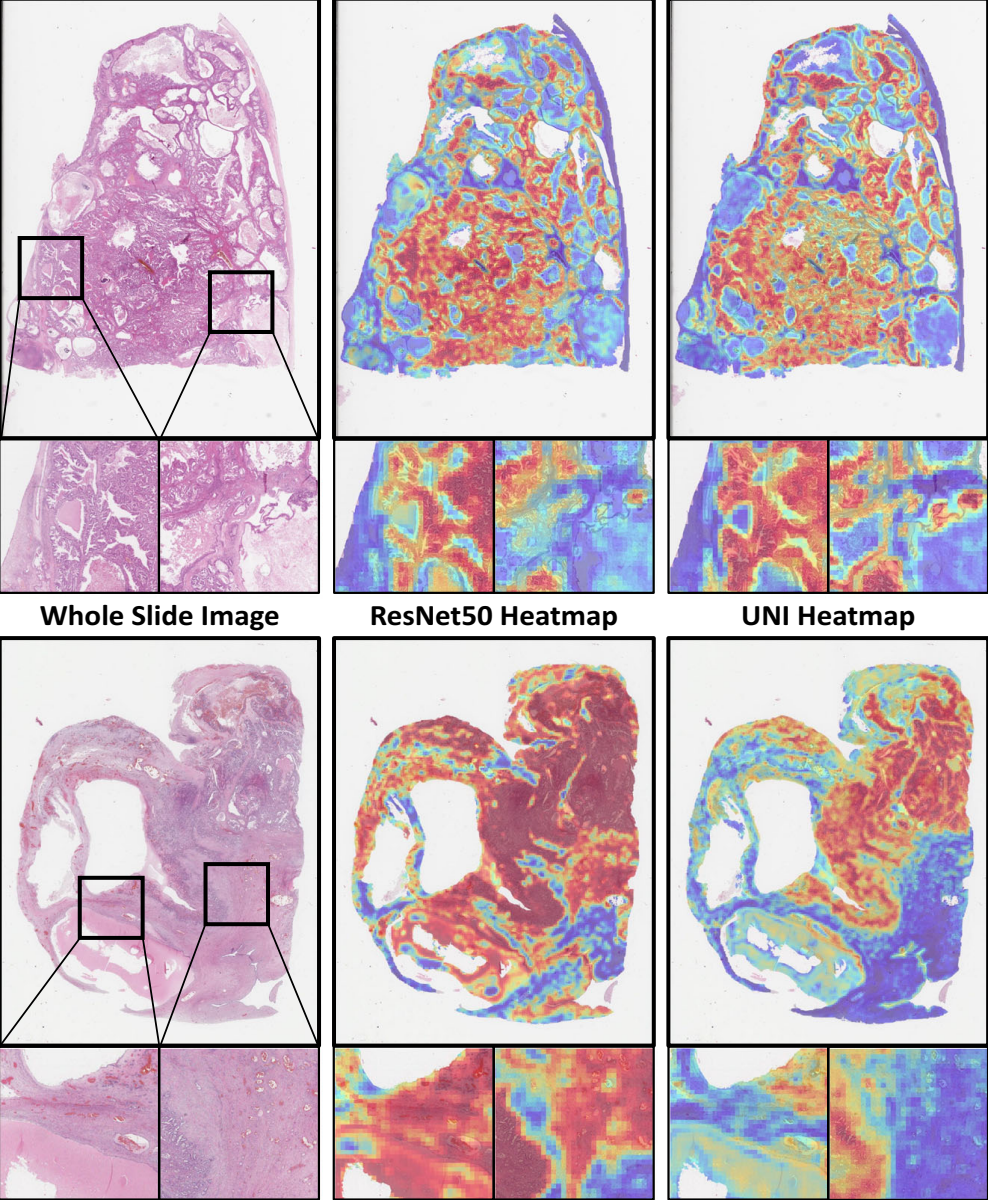
Attention heatmaps. Example attention heatmaps from the ABMIL classifier using the ImageNet-pretrained ResNet50 and UNI foundation model features. (Upper) A typical difference between heatmaps with different diagnoses. (Lower) The most extreme qualitative difference found between heatmaps in the internal test set. In both examples, the UNI classification was correct (upper—MC, lower—CCC), and the ResNet50 classification was incorrect (upper—EC, lower—MC). These heatmaps are based on 256 × 256 pixel patches with 50% overlap at 10× apparent magnification, with visual differences in scale caused by the variable size of resection samples.

To qualitatively analyse the differences between foundation models and ImageNet-pretrained CNNs, two pathologists (K.A. and N.M.O.) compared the ABMIL attention heatmaps (Fig. 9) generated using the baseline ResNet50 and the UNI foundation model. Most heatmaps were well-focused on tumour and relevant stromal regions for both models, with often only subtle differences between them. The UNI-based heatmaps generally indicated a slightly greater focus on tumour tissue, whereas the ResNet50 model also paid attention to some stromal regions of variable diagnostic relevance (Supplementary Figs. 5, 6). Attention heatmaps can be useful for identifying potential sources of error but should be interpreted with caution since they cannot provide a complete explanation of classification decisions^66^.

All of the WSIs which were misclassified by the optimal H-optimus-0 model (Fig. 3) in hold-out testing were reviewed by the pathologists involved in the study, who found that the majority (6/11) had incorrect ground-truth labels, and had been correctly classified by the model. This underscores the value of the model in detecting the human errors which occur in the production of large-scale repositories. A subsequent review to identify any possible further labelling errors affecting internal data did not locate any issues. The five slides that were truly misclassified by the model in hold-out testing (three CCC classified as HGSC, one CCC classified as LGSC, and one LGSC classified as EC) showed the typical morphology (both architectural and cytological) of their true subtypes, making it unclear why these errors occurred.

From cross-validation, a selection of misclassified slides was reviewed by the pathology team. The 42 EC slides classified as other subtypes all exhibited potentially confusing morphological features that occur within the spectrum of EC, including villoglandular and papillary architecture as well as foci of mucinous and squamous metaplastic differentiation, and squamous morule formation. ECs misclassified as HGSCs were of a higher grade and featured both greater nuclear pleomorphism and a more solid growth pattern. It would be interesting to determine whether any of these misclassifications are reflective of shared underlying molecular aberrations, which will form the focus of our future work. The most commonly confused subtypes were HGSC and LGSC, which is not surprising considering their similar histoarchitecture. These entities were historically considered a single entity with a three-tier grading system until the characterisation of their distinct molecular alterations and clinical behaviours^67^. Collecting additional training data may help to improve the discrimination of these similar subtypes, with LGSC having only formed 5% of the training set due to it being a relatively uncommon subtype.

The strong performance of foundation models in this study was particularly impressive considering that they were applied here at 10× magnification, despite often only being trained using 20× magnification data. This was a practical computational limitation when performing hyperparameter tuning, as 20× magnification tissue would produce approximately 4 times as many patches per WSI as 20× magnification tissue, thus quadrupling the total runtime. While 10× magnification was previously found to be best for this task when using the ImageNet-pretrained ResNet50^14^, it may not have been optimal when using foundation models that had typically been trained at 20× magnification. However, a previous study of foundation models for slide-level classification found no consistent benefit from increasing to 20× magnification^64^.

In this study, we reported the second-highest-ever performance of an AI model for ovarian cancer subtyping (behind our concurrent study using multi-resolution graph networks with the UNI encoder^15^), with 97% balanced accuracy on the Transcanadian Study dataset. However, results were variable across datasets. The improved performance from histopathology foundation models is promising for the potential clinical utility of these AI approaches, though further work is required to ensure that the models generalise to all relevant sources of variation, especially across different histopathology labs and slide scanners. This may require larger, more diverse training datasets. Models should be made robust to the influence of lower-quality data and artefacts to reduce the burden of quality control. Ideally, models should also be able to accurately classify post-treatment tissue, though if this proves infeasible, it may be necessary to restrict the scope of the models to the classification of high-quality primary surgery tissue samples, for which these models already excel. Furthermore, it is currently unclear how best to present automatically generated information to pathologists to assist them, rather than to distract, frustrate, or confuse them. This may require improved model interpretability and a measure of model uncertainty, especially considering the existence of rare subtypes which are notoriously difficult to collect sufficient data on outside the context of multi-centre collections.

Ideally, algorithms would be made more computationally efficient for use in the clinic, but the best-performing foundation models are less computationally efficient than the ResNet CNN. This problem is exacerbated by the limited digitisation of histopathology services, with most pathological diagnoses still made under a microscope. AI adoption will be contingent on it being accessible and beneficial given the limited computational infrastructure and users who may not be technological experts. While various issues are inhibiting the clinical translation of ovarian cancer subtyping models, these seem increasingly likely to be overcome in the near future.

In this study, we conducted a rigorous validation of feature extraction methods for ovarian cancer subtyping. We found that the features generated by histopathology foundation models drastically improved downstream classification performance when compared to ImageNet-pretrained feature extractors. Several different data preprocessing techniques were evaluated in an attempt to improve the performance of the ImageNet-pretrained ResNet50 baseline, and while these somewhat improved performance, they were far from sufficient to match the performance of the foundation models. Through a five-fold ensemble of ABMIL classifiers, the best overall foundation model, H-optimus-0, achieved a five-class balanced accuracy of 89% on internal test data and 97% and 80% on external test sets, compared to 68%, 81%, and 55% respectively for the best ImageNet-pretrained ResNet models. This represents the greatest performance for the ovarian carcinoma subtype classification task in any peer-reviewed literature to date. The largest models and those pretrained with the largest datasets generally gave the best performance, though the UNI foundation model was one of the best-performing models despite a relatively moderate pretraining dataset and model size, giving an average balanced accuracy of only 0.1% lower than H-optimus-0 while running over 4 times as fast. Hyperparameter tuning the downstream classifiers improved classification performance by a median of 1.9% balanced accuracy, although this was variable. While the improved classification performance offered by histopathology foundation models may be sufficient for clinical implementation, the need to address logistical hurdles and conduct larger-scale validations remains.

## Supplementary information

Supplementary information

## Data Availability

This study was conducted retrospectively using human subject data and received approval from the Wales Research Ethics Committee [18/WA/0222] and the Confidentiality Advisory Group [18/CAG/0124]. Approval has not yet been provided for this data to be shared outside of the research group. The Transcanadian Study dataset was downloaded from https://www.medicalimageanalysis.com/data/ovarian-carcinomas-histopathology-dataset (last accessed 09/04/24). The OCEAN Challenge dataset was downloaded from https://www.kaggle.com/competitions/UBC-OCEAN/data (last accessed 20/08/24).
