# Supplementary material for: A comprehensive evaluation of histopathology foundation models for ovarian cancer subtype classification
Source: NPJ Precis Oncol. 2025 Jan 30;9:33. doi: 10.1038/s41698-025-00799-8 (PMC11782474; doi:10.1038/s41698-025-00799-8)
Supplement: Supplementary file 1 — Supplementary information [file 41698_2025_799_MOESM1_ESM.pdf]

SUPPLEMENTARY MATERIALS

A HYPERPARAMETER TUNING DETAILS

Tuning Iteration	Learning Rate	Weight Decay	First Moment Decay	Second Moment Decay	Stability Parameter	LR Decay Patience	LR Decay Factor	Model Size	Drop Out	Max Patches
1	✓							✓		
2									✓	✓
3			✓	✓						
4	✓	✓								
5			✓		✓					
6								✓		✓
7						✓	✓			
8	✓								✓	
9								✓		
10	✓					✓		✓		
11									✓	✓
12						✓	✓			
13	✓							✓		
14		✓								✓
15								✓		
16			✓	✓						
17	✓		✓					✓	✓	✓

Supplementary Table 1. Hyperparameter tuning iterations. Check marks (✓) indicate the hyperparameters that were adjusted at each stage of tuning, with all others frozen.

Hyperparameter tuning was conducted over seventeen iterations for the ten ABMIL classifier hyperparameters, as shown in Supplementary Table 1. The optimal hyperparameters (Supplementary Table 2) typically did not vary greatly for models using the same feature extraction backbone, with a few notable exceptions. The regularisation hyperparameters (drop out, weight decay, max patches) varied greatly across all models, including those with the same backbone. The classifier based on the x5 augmented training data was the smallest ResNet50-based classifier by far (and had the smallest stability parameter and learning rate decay factor), with only 0.1M parameters compared to the next smallest at 0.7M. The ViT-based models had between 0.2M (H-optimus-0) and 1.6M parameters (Virchow). The largest ViT-based encoders typically had smaller values for the first moment decay (0.5-0.75) than the smaller ViT-based encoders (0.9-0.99). Other hyperparameters were relatively stable within a given feature extractor.

Some hyperparameters varied greatly between model architectures. The learning rate was much smaller for ViT-based models (0.00001-0.0002) than ImageNet-pretrained ResNet50 models (0.001-0.002) and often had a faster rate of decay. The Adam optimiser first and second moment decay parameters were also often higher in ViT-based models than in ResNet50 models. Other hyperparameters were relatively consistent between model architectures.

As shown in Figure 6, most of the benefit of hyperparameter tuning was achieved within the first few tuning iterations. This indicates the importance of tuning the key hyperparameters (especially learning rate and model size) and also indicates that hyperparameter tuning does not necessarily need to be extensive to provide a benefit. The models with larger feature extractors typically achieved smaller loss values in tuning. It is worth noting that the ABMIL classifiers were orders of magnitude smaller than the

feature extraction models, making it much more computationally feasible to tune the classifiers than the feature extractors.

Feature Extractor	Learning Rate (LR)	LR Decay Patience	LR Decay Factor	First Moment Decay	Second Moment Decay	Stability Parameter	Drop Out	Weight Decay	Max Patches	Model Size
RN50	2e-3	20	0.75	0.75	0.95	1e-2	0.4	1e-3	800	[512,128]
RN18	1e-4	20	0.9	0.8	0.99	1e-4	0.5	1e-5	700	[1024,256]
ViT-L	5e-5	10	0.35	0.85	0.999	1e-3	0.0	1e-1	800	[512,384]
RN18-Histo	2e-4	20	0.9	0.9	0.99	1e-4	0.6	1e-4	1000	[512,512]
Lunit	1e-4	10	0.75	0.99	0.9999	1e-5	0.6	1e-1	900	[1024,512]
RN50-Histo	2e-4	25	0.75	0.8	0.99	1e-4	0.6	1e-3	700	[512,384]
CTransPath	1e-4	25	0.9	0.7	0.99999	1e-3	0.4	1e-3	1000	[256,128]
Hibou-B	4e-5	10	0.9	0.99	0.9999	1e-3	0.3	1e-2	1600	[256,128]
Phikon	5e-5	25	0.75	0.99	0.999	1e-5	0.8	1e-5	1200	[512,256]
Kaiko-B8	2e-5	10	0.75	0.95	0.9999	1e-5	0.2	1e-1	600	[512,128]
GPFM	1e-4	25	0.9	0.95	0.99	1e-4	0.8	1e-6	1000	[512,128]
UNI	1e-5	10	0.75	0.9	0.999	1e-5	0.0	1e-3	1000	[512,256]
Hibou-L	5e-5	25	0.75	0.75	0.99999	1e-4	0.6	1e-7	400	[256,128]
Virchow	2e-4	20	0.9	0.95	0.99	1e-3	0.8	1e-2	1100	[512,256]
Virchow2-CLS	2e-5	10	0.75	0.55	0.999	1e-4	0.6	1e-4	1000	[512,256]
H-optimus-0	2.5e-5	5	0.75	0.5	0.9999	1e-4	0.4	1e-2	1000	[128,32]
Prov-GigaPath	5e-5	15	0.75	0.7	0.99	1e-4	0.7	1e-4	1300	[512,256]
RN50 Reinhard	2e-3	25	0.75	0.75	0.95	1e-2	0.4	1e-3	400	[512,256]
RN50 Macenko	2e-3	15	0.75	0.85	0.95	1e-2	0.3	1e-3	400	[512,128]
RN50 Otsu	2e-3	15	0.9	0.75	0.95	1e-2	0.1	1e-3	600	[512,256]
RN50 Otsu+Macenko	2e-3	25	0.9	0.75	0.99	1e-3	0.3	1e-4	1000	[512,256]
RN50 5Augs	1e-3	25	0.6	0.8	0.99	1e-4	0.4	1e-4	700	[128,32]
RN50 10Augs	2e-3	20	0.75	0.8	0.99	1e-2	0.4	1e-3	700	[512,256]
RN50 20Augs	1e-3	20	0.75	0.7	0.999	1e-3	0.6	1e-4	1000	[512,128]

Supplementary Table 2. Optimal hyperparameters. The final hyperparameters of each model determined by an iterative grid search tuning procedure using five cross-validation folds, including the models from the ablation study. The model size is presented as the number of parameters in the attention layer and subsequent fully connected layer.

B EXPANDED RESULTS

Feature Extractor	Balanced Accuracy	AUROC	F1 Score
RN50	57.1% (53.8-60.4%)	0.893 (0.879-0.907)	0.596 (0.561-0.630)
RN18	56.1% (52.8-59.4%)	0.882 (0.866-0.898)	0.584 (0.551-0.617)
ViT-L	62.6% (59.2-66.0%)	0.893 (0.877-0.909)	0.628 (0.596-0.660)
RN18-Histo	59.1% (55.8-62.4%)	0.887 (0.871-0.902)	0.615 (0.582-0.648)
Lunit	66.6% (63.3-70.0%)	0.910 (0.894-0.926)	0.682 (0.649-0.714)
RN50-Histo	62.4% (59.2-65.6%)	0.925 (0.911-0.938)	0.651 (0.618-0.684)
CTransPath	67.3% (63.9-70.6%)	0.925 (0.912-0.938)	0.669 (0.638-0.700)
Hibou-B	67.7% (64.4-71.0%)	0.945 (0.935-0.954)	0.689 (0.656-0.720)
Phikon	67.0% (63.7-70.4%)	0.926 (0.912-0.938)	0.684 (0.653-0.715)
Kaiko-B8	70.3% (67.0-73.6%)	0.933 (0.919-0.946)	0.720 (0.688-0.751)
GPFM	70.9% (67.7-74.1%)	0.935 (0.923-0.948)	0.710 (0.680-0.739)
UNI	73.2% (69.9-76.4%)	0.945 (0.933-0.956)	0.734 (0.704-0.764)
Hibou-L	67.0% (63.6-70.3%)	0.930 (0.918-0.942)	0.690 (0.656-0.721)
Virchow	68.6% (65.3-71.8%)	0.936 (0.925-0.947)	0.688 (0.658-0.717)
Virchow2-CLS	**74.7%** (71.5-77.9%)	0.943 (0.930-0.954)	**0.742** (0.713-0.771)
H-optimus-0	72.2% (68.9-75.4%)	**0.947** (0.936-0.957)	0.726 (0.695-0.756)
Prov-GigaPath	71.2% (67.9-74.4%)	0.927 (0.913-0.941)	0.725 (0.696-0.754)

Supplementary Table 3. Results of five-fold cross-validation. Results are reported as the mean and 95% confidence intervals (in brackets) from 10,000 iterations of bootstrapping. The greatest results are shown in **bold**.

Feature Extractor	Balanced Accuracy	AUROC	F1 Score
RN50	66.0% (58.1-73.7%)	0.916 (0.873-0.953)	0.634 (0.537-0.726)
RN18	64.0% (55.3-72.6%)	0.930 (0.893-0.963)	0.628 (0.530-0.723)
ViT-L	76.0% (67.8-83.7%)	0.926 (0.885-0.963)	0.747 (0.656-0.832)
RN18-Histo	65.0% (57.1-72.5%)	0.890 (0.843-0.932)	0.613 (0.531-0.698)
Lunit	79.1% (71.4-86.3%)	0.943 (0.904-0.977)	0.778 (0.693-0.857)
RN50-Histo	74.1% (65.7-81.9%)	0.946 (0.908-0.977)	0.730 (0.641-0.815)
CTransPath	81.0% (74.0-88.0%)	0.950 (0.911-0.982)	0.797 (0.716-0.873)
Hibou-B	87.0% (81.0-92.6%)	0.956 (0.921-0.985)	0.858 (0.783-0.925)
Phikon	79.0% (72.0-85.7%)	0.946 (0.907-0.979)	0.772 (0.689-0.852)
Kaiko-B8	83.0% (75.8-89.9%)	0.947 (0.909-0.980)	0.823 (0.746-0.896)
GPFM	82.0% (74.8-88.7%)	0.955 (0.918-0.985)	0.809 (0.728-0.884)
UNI	88.0% (81.5-93.8%)	0.957 (0.919-0.989)	0.875 (0.805-0.937)
Hibou-L	82.1% (75.5-88.4%)	0.959 (0.921-0.990)	0.804 (0.722-0.880)
Virchow	85.0% (78.4-91.1%)	**0.964** (0.928-0.993)	0.839 (0.763-0.909)
Virchow2-CLS	88.0% (81.9-93.8%)	**0.964** (0.926-0.994)	0.873 (0.802-0.937)
H-optimus-0	**89.0%** (83.1-94.3%)	0.963 (0.925-0.992)	**0.883** (0.815-0.944)
Prov-GigaPath	84.0% (77.4-90.3%)	0.958 (0.924-0.986)	0.830 (0.752-0.900)

Supplementary Table 4. Results of hold-out testing. Predictions were generated by an ensemble of the five-fold classification models. Results are reported as the mean and 95% confidence intervals (in brackets) from 10,000 iterations of bootstrapping. The greatest results are shown in **bold**.

Feature Extractor	Balanced Accuracy	AUROC	F1 Score
RN50	69.2% (58.7-79.7%)	0.956 (0.928-0.980)	0.696 (0.582-0.807)
RN18	79.0% (68.8-88.6%)	0.959 (0.923-0.985)	0.804 (0.700-0.896)
ViT-L	80.7% (72.2-89.2%)	0.970 (0.937-0.993)	0.814 (0.712-0.908)
RN18-Histo	66.5% (55.2-77.5%)	0.930 (0.888-0.965)	0.653 (0.539-0.763)
Lunit	95.0% (89.3-99.1%)	0.998 (0.994-1.000)	0.930 (0.862-0.985)
RN50-Histo	94.4% (88.2-98.9%)	0.994 (0.985-0.999)	0.934 (0.870-0.985)
CTransPath	88.8% (80.9-95.6%)	0.982 (0.959-0.996)	0.861 (0.773-0.939)
Hibou-B	91.1% (83.0-97.9%)	0.990 (0.979-0.998)	0.921 (0.850-0.979)
Phikon	90.3% (81.9-97.8%)	0.994 (0.986-0.999)	0.919 (0.839-0.982)
Kaiko-B8	96.7% (93.8-99.2%)	0.997 (0.991-1.000)	0.937 (0.879-0.986)
GPFM	**98.3%** (95.6-100.0%)	**0.999** (0.997-1.000)	**0.977** (0.937-1.000)
UNI	93.2% (86.5-98.3%)	0.996 (0.988-1.000)	0.912 (0.835-0.974)
Hibou-L	89.3% (80.6-96.2%)	0.989 (0.975-0.998)	0.889 (0.805-0.959)
Virchow	87.5% (79.0-94.8%)	0.993 (0.984-0.999)	0.848 (0.750-0.931)
Virchow2-CLS	88.0% (79.8-95.3%)	0.997 (0.993-1.000)	0.871 (0.779-0.952)
H-optimus-0	96.7% (91.1-100.0%)	**0.999** (0.998-1.000)	0.975 (0.931-1.000)
Prov-GigaPath	88.6% (80.2-95.9%)	0.995 (0.987-1.000)	0.878 (0.783-0.958)

Supplementary Table 5. Results of external validation on the Transcanadian Study dataset. Predictions were generated by an ensemble of the five-fold classification models. Results are reported as the mean and 95% confidence intervals (in brackets) from 10,000 iterations of bootstrapping. The greatest results are shown in **bold**.

Feature Extractor	Balanced Accuracy	AUROC	F1 Score
RN50	52.4% (49.5-55.1%)	0.868 (0.847-0.889)	0.412 (0.380-0.444)
RN18	51.9% (48.6-54.9%)	0.841 (0.820-0.863)	0.412 (0.377-0.448)
ViT-L	59.5% (55.4-63.6%)	0.880 (0.857-0.902)	0.578 (0.532-0.625)
RN18-Histo	57.3% (54.0-60.4%)	0.850 (0.828-0.872)	0.523 (0.484-0.563)
Lunit	73.6% (69.7-77.5%)	0.954 (0.941-0.967)	0.729 (0.681-0.775)
RN50-Histo	68.0% (64.5-71.6%)	0.946 (0.930-0.959)	0.679 (0.634-0.725)
CTransPath	67.8% (64.0-71.7%)	0.934 (0.917-0.950)	0.676 (0.629-0.724)
Hibou-B	65.4% (61.4-69.4%)	0.935 (0.920-0.949)	0.633 (0.582-0.682)
Phikon	66.4% (62.8-70.1%)	0.898 (0.879-0.917)	0.642 (0.595-0.689)
Kaiko-B8	70.0% (65.4-74.5%)	0.941 (0.925-0.956)	0.695 (0.644-0.744)
GPFM	74.5% (70.4-78.5%)	0.935 (0.919-0.949)	0.746 (0.702-0.788)
UNI	77.2% (73.0-81.4%)	0.954 (0.939-0.966)	0.758 (0.714-0.801)
Hibou-L	69.3% (66.2-72.3%)	0.946 (0.931-0.959)	0.663 (0.622-0.706)
Virchow	79.2% (75.2-83.0%)	**0.959** (0.946-0.970)	**0.765** (0.722-0.807)
Virchow2-CLS	**79.8%** (75.8-83.6%)	0.958 (0.945-0.970)	0.759 (0.717-0.801)
H-optimus-0	74.0% (69.9-78.1%)	0.952 (0.939-0.963)	0.703 (0.656-0.748)
Prov-GigaPath	75.4% (71.3-79.3%)	**0.959** (0.946-0.970)	0.729 (0.684-0.771)

Supplementary Table 6. Results of external validation on the OCEAN dataset. Predictions were generated by an ensemble of the five-fold classification models. Results are reported as the mean and 95% confidence intervals (in brackets) from 10,000 iterations of bootstrapping. The greatest results are shown in **bold**.

	Subtype	F1 Score	Precision	Recall / Sensitivity	Specificity	Balanced Accuracy
Cross-Validation	HGSC	0.925	0.925	0.926	0.841	0.883
	LGSC	0.443	0.405	0.489	0.963	0.726
	CCC	0.814	0.832	0.798	0.981	0.889
	EC	0.782	0.766	0.799	0.969	0.884
	MC	0.667	0.756	0.596	0.989	0.793
Hold-out Testing	HGSC	0.870	0.769	1.000	0.925	0.963
	LGSC	0.865	0.941	0.800	0.988	0.894
	CCC	0.765	0.929	0.650	0.988	0.819
	EC	0.976	0.952	1.000	0.988	0.994
	MC	0.952	0.909	1.000	0.975	0.988
Transcanadian Study	HGSC	0.968	0.938	1.000	0.960	0.980
	LGSC	0.941	1.000	0.889	1.000	0.944
	CCC	0.974	1.000	0.950	1.000	0.975
	EC	1.000	1.000	1.000	1.000	1.000
	MC	1.000	1.000	1.000	1.000	1.000
OCEAN Challenge	HGSC	0.807	0.813	0.802	0.865	0.833
	LGSC	0.582	0.622	0.548	0.970	0.759
	CCC	0.777	0.635	1.000	0.871	0.936
	EC	0.606	0.946	0.445	0.992	0.719
	MC	0.747	0.638	0.902	0.956	0.929

Supplementary Table 7. Classwise metrics. Additional classwise classification metrics for the optimal H-optimus-0 ABMIL classifier.

Cross-validation – IDS Samples

Predicted Subtype (columns); Actual Subtype (rows)

	HGSC	LGSC	CCC	EC	MC
**HGSC**	**348**	23	12	7	1
**LGSC**	12	**22**	1	0	0
**CCC**	5	1	**15**	0	0
**EC**	3	0	0	**0**	0
**MC**	0	0	0	0	**2**

Cross-validation – Staging Samples

Predicted Subtype (columns); Actual Subtype (rows)

	HGSC	LGSC	CCC	EC	MC
**HGSC**	**824**	29	11	10	1
**LGSC**	30	**23**	3	1	0
**CCC**	27	6	**143**	0	1
**EC**	16	7	0	**167**	16
**MC**	2	0	5	33	**57**

Supplementary Figure 1. Results by treatment status. Confusion matrices for the optimal ABMIL classifier with features from the H-optimus-0 foundation model in cross-validation broken down by treatment status. Correct classifications are indicated in green.

C HYPERPARAMETER TUNING ABLATION

The results of each model using the default hyperparameters (without tuning) are shown in Supplementary Tables 8-11, with indications about whether the default hyperparameters improved or degraded performance compared to using the tuned hyperparameters. Exact p-values are provided in Section E.

Feature Extractor	Balanced Accuracy		AUROC		F1 Score	
RN50	57.5%	-	0.877	↓	0.593	-
RN18	55.3%	-	0.857	↓	0.561	↓
ViT-L	56.2%	↓↓↓*	0.857	↓↓*	0.580	↓↓*
RN18-Histo	56.0%	↓↓*	0.879	-	0.574	↓↓*
Lunit	65.3%	↓	0.891	↓*	0.646	↓↓
RN50-Histo	63.1%	-	0.915	↓	0.656	-
CTransPath	68.8%	↑	0.927	-	0.690	↑
Hibou-B	66.1%	↓	0.911	↓↓	0.667	↓
Phikon	68.0%	↑	0.912	↓	0.672	↓
Kaiko-B8	62.7%	↓↓↓*	0.907	↓	0.633	↓↓↓*
GPFM	69.4%	↓	0.912	↓*	0.690	↓
UNI	67.1%	↓↓↓*	0.915	↓↓*	0.684	↓↓↓*
Hibou-L	58.7%	↓↓↓	0.889	↓↓*	0.622	↓↓↓
Virchow	65.2%	↓↓*	0.896	↓↓	0.658	↓↓
Virchow2-CLS	69.8%	↓↓	0.917	↓*	0.681	↓↓↓
H-optimus-0	66.1%	↓↓↓	0.916	↓↓	0.678	↓↓
Prov-GigaPath	67.9%	↓↓	0.919	-	0.675	↓↓↓

Supplementary Table 8. Results of five-fold cross-validation without hyperparameter tuning. Arrows indicate the absolute difference in performance compared to the tuned models, with one arrow (↑) for difference a of at least 1%, two arrows (↑↑) for a difference of at least 3%, and three arrows (↑↑↑) for a difference of at least 5%. *Indicates a p-value less than 0.05 when comparing to the tuned model.

Feature Extractor	Balanced Accuracy		AUROC		F1 Score	
RN50	68.0%	↑	0.923	-	0.670	↑↑
RN18	64.0%	-	0.927	-	0.626	-
ViT-L	61.0%	↓↓↓*	0.917	-	0.601	↓↓↓*
RN18-Histo	62.1%	↓	0.889	-	0.586	↓
Lunit	74.0%	↓↓↓	0.932	↓	0.727	↓↓↓
RN50-Histo	74.9%	-	0.943	-	0.739	-
CTransPath	78.0%	↓↓*	0.941	-	0.768	↓*
Hibou-B	78.0%	↓↓↓*	0.958	-	0.765	↓↓↓*
Phikon	80.1%	↑	0.941	-	0.792	↑
Kaiko-B8	79.0%	↓↓	0.949	-	0.786	↓↓
GPFM	84.0%	↑	0.953	-	0.831	↑
UNI	82.0%	↓↓↓*	0.962	-	0.806	↓↓↓*
Hibou-L	75.0%	↓↓↓*	0.959	-	0.730	↓↓↓*
Virchow	81.0%	↓↓	0.956	-	0.801	↓↓
Virchow2-CLS	89.1%	↑	0.963	-	0.883	↑
H-optimus-0	85.0%	↓↓	0.965	-	0.843	↓↓
Prov-GigaPath	83.0%	↓	0.949	-*	0.820	↓

Supplementary Table 9. Results of hold-out testing without hyperparameter tuning. Arrows indicate the absolute difference in performance compared to the tuned models, with one arrow (↑) for difference a of at least 1%, two arrows (↑↑) for a difference of at least 3%, and three arrows (↑↑↑) for a difference of at least 5%. *Indicates a p-value less than 0.05 when comparing to the tuned model. While the Prov-GigaPath AUROC only exhibited a reduction of 0.009, this was found to be statistically significant.

Feature Extractor	Balanced Accuracy		AUROC		F1 Score	
RN50	75.8%	↑↑↑	0.969	↑↑	0.769	↑↑↑
RN18	80.1%	↑	0.946	↓	0.807	-
ViT-L	68.9%	↓↓↓*	0.960	↓	0.702	↓↓↓*
RN18-Histo	69.3%	↑	0.942	↑	0.689	↑↑
Lunit	92.4%	↓	0.989	-	0.879	↓↓↓
RN50-Histo	91.8%	↓	0.995	-	0.902	↓↓
CTransPath	88.1%	-	0.978	-	0.847	↓
Hibou-B	85.3%	↓↓↓	0.987	-	0.871	↓↓↓
Phikon	94.6%	↑↑	0.996	-	0.944	↑
Kaiko-B8	93.3%	↓↓	0.996	-	0.926	↓
GPFM	97.7%	-	0.998	-	0.964	↓
UNI	95.3%	↑	0.996	-	0.952	↑↑
Hibou-L	81.7%	↓↓↓	0.988	-*	0.813	↓↓↓*
Virchow	88.8%	↑	0.991	-	0.864	↑
Virchow2-CLS	91.6%	↑↑	1.000	-	0.915	↑↑
H-optimus-0	99.0%	↑	1.000	-	0.991	↑
Prov-GigaPath	89.4%	-	0.993	-	0.871	-

Supplementary Table 10. Results of validation on the Transcanadian Study dataset without hyperparameter tuning. Arrows indicate the absolute difference in performance compared to the tuned models, with one arrow (↑) for difference a of at least 1%, two arrows (↑↑) for a difference of at least 3%, and three arrows (↑↑↑) for a difference of at least 5%. *Indicates a p-value less than 0.05 when comparing to the tuned model.

Feature Extractor	Balanced Accuracy		AUROC		F1 Score	
RN50	51.0%	↓	0.857	↓	0.411	-
RN18	49.0%	↓	0.837	-	0.370	↓↓
ViT-L	48.3%	↓↓↓*	0.843	↓↓*	0.506	↓↓↓*
RN18-Histo	55.0%	↓	0.849	-	0.504	↓
Lunit	68.8%	↓↓	0.935	↓*	0.661	↓↓↓
RN50-Histo	66.6%	↓	0.934	↓*	0.684	-
CTransPath	68.1%	-	0.934	-	0.686	↑
Hibou-B	64.0%	↓	0.928	-	0.604	↓
Phikon	63.2%	↓↓	0.903	-	0.598	↓↓
Kaiko-B8	64.1%	↓↓↓	0.929	↓*	0.596	↓↓*
GPFM	73.8%	-	0.937	-	0.725	↓
UNI	69.6%	↓↓↓*	0.948	-*	0.693	↓↓↓*
Hibou-L	64.7%	↓↓	0.936	↓*	0.615	↓↓
Virchow	78.5%	-*	0.948	↓*	0.766	-
Virchow2-CLS	74.0%	↓↓↓*	0.956	-	0.719	↓↓*
H-optimus-0	74.8%	-	0.951	-	0.747	↑↑
Prov-GigaPath	75.4%	-	0.957	-	0.720	-

Supplementary Table 11. Results of validation on the OCEAN Challenge dataset without hyperparameter tuning. Arrows indicate the absolute difference in performance compared to the tuned models, with one arrow (↑) for difference a of at least 1%, two arrows (↑↑) for a difference of at least 3%, and three arrows (↑↑↑) for a difference of at least 5%. *Indicates a p-value less than 0.05 when comparing to the tuned model. The UNI AUROC only exhibited a reduction of 0.006, but this was found to be statistically significant. The Virchow balanced accuracy only exhibited a reduction of 0.7%, but this was found to be statistically significant.

D AUGMENTATION AND NORMALISATIONS

Preprocessing techniques were compared using the baseline ImageNet-pretrained ResNet50 encoder with the default feature extraction settings from the CLAM repository [1] (static saturation thresholding with no augmentation or normalisation). Comparisons were made using Reinhard normalisation, Macenko normalisation, Otsu thresholding, Otsu thresholding with Macenko normalisation, and colour augmentations to increase the effective training set size by factors of 5×, 10×, and 20×.

Otsu thresholding [2] is applied during tissue detection to automatically determine the saturation threshold for each image by minimising the variance within the separated high-saturation and low-saturation groups. Saturation thresholding is a computationally efficient tissue segmentation approach, but risks including artifacts such as bubbles, pen marks, and coverslip edges in the foreground. While more robust (and complex) tissue segmentation techniques exist [3, 4], we focused on simple approaches as the attention mechanism in the classification models should learn to ignore any remaining artifacts. We compared the CLAM [1] default static threshold (8/255) to Otsu thresholding with parameters manually adjusted to qualitatively improve the segmentation.

Normalisation and augmentation techniques control data variability, which is particularly important for generalisability in histopathology, where varied staining and scanning procedures between labs result in chromatic differences [5]. Normalisation reduces variability, adjusting images into a consistent colour space to allow models to learn general features. We investigated two commonly used [6] stain normalisation techniques - Reinhard normalisation [7] and Macenko normalisation [8]. These approaches work in logarithmic colour spaces, where stains behave linearly, making them easier to separate and manipulate. Reinhard normalisation is a standard normalisation technique applied in $l\alpha\beta$ space (radiance l, blue-yellow α, red-green β). Macenko normalisation uses singular value decomposition to separate stain and saturation values, before scaling stain values in logarithmic RGB space. Basic RGB normalisations were also applied to all images (after any other colour adjustments) to match the ImageNet and histopathology-specific pretraining procedures. While many more sophisticated stain normalisation techniques have been developed, it is unclear whether any such approach is better than Macenko normalisation overall [6].

Augmentation techniques conversely increase the variability of the training data to allow the model to learn a more general domain. For such large images, training models end-to-end to allow for online data augmentation (adjustments during training) is extremely computationally intensive [9]. Some researchers have attempted to apply online augmentations in the embedding space using generative models [10, 11], though this adds an extra layer of complexity to an already resource-intensive model pipeline. Instead, offline augmentation creates a finite set of augmented versions of the original data, artificially increasing the diversity of training data to a lesser extent than online augmentation. We investigated colour augmentations which adjusted the brightness, contrast, saturation and hue of each patch using parameters from a previous study [12], which we found to create plausibly altered colours (Supplementary Figure 2).

Extended Results

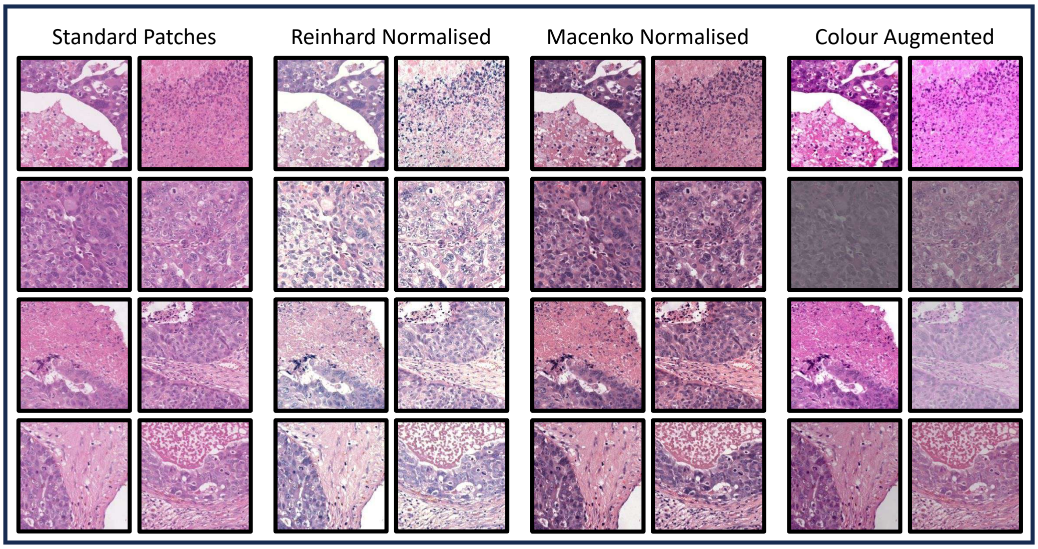

Supplementary Figure 2. Tissue normalisation and augmentation procedures. Illustrated using 256x256 pixel patches from a single whole slide image at 10× magnification.

Preprocessing Approach	Balanced Accuracy	AUROC	F1 Score
Baseline	57.1% (53.8-60.4%)	**0.893** (0.879-0.907)	0.596 (0.561-0.630)
Reinhard Normalisation	51.3% (48.2-54.4%)	0.872 (0.856-0.887)	0.520 (0.488-0.553)
Macenko Normalisation	57.8% (54.5-61.2%)	0.882 (0.867-0.896)	0.601 (0.567-0.635)
Otsu Thresholding	53.9% (50.6-57.2%)	0.888 (0.873-0.903)	0.566 (0.532-0.600)
Otsu + Macenko	58.0% (54.6-61.4%)	0.882 (0.865-0.898)	0.605 (0.571-0.638)
5× Colour Augmentation	57.4% (54.0-60.7%)	0.888 (0.873-0.902)	0.592 (0.560-0.625)
10× Colour Augmentation	**59.1%** (55.7-62.4%)	0.891 (0.877-0.905)	**0.615** (0.581-0.649)
20× Colour Augmentation	**59.1%** (55.7-62.4%)	0.892 (0.877-0.905)	0.596 (0.564-0.627)

Supplementary Table 12. Results of five-fold cross-validation for the ImageNet-pretrained ResNet50 with varied preprocessing approaches. Results are reported as the mean and 95% confidence intervals (in brackets) from 10,000 iterations of bootstrapping. The greatest results are shown in **bold**.

Preprocessing Approach	Balanced Accuracy	AUROC	F1 Score
Baseline	66.0% (58.1-73.7%)	0.916 (0.873-0.953)	0.634 (0.537-0.726)
Reinhard Normalisation	65.0% (56.6-73.2%)	**0.923** (0.881-0.961)	0.632 (0.534-0.727)
Macenko Normalisation	63.0% (54.4-71.5%)	0.915 (0.873-0.951)	0.620 (0.521-0.715)
Otsu Thresholding	65.0% (56.7-73.4%)	0.916 (0.872-0.955)	0.637 (0.542-0.732)
Otsu + Macenko	59.0% (50.3-67.6%)	0.918 (0.878-0.952)	0.577 (0.475-0.674)
5× Colour Augmentation	65.0% (57.0-72.9%)	0.916 (0.876-0.951)	0.630 (0.536-0.725)
10× Colour Augmentation	64.0% (55.9-72.1%)	0.906 (0.864-0.944)	0.616 (0.522-0.710)
20× Colour Augmentation	**68.0%** (59.7-76.0%)	0.904 (0.861-0.942)	**0.657** (0.563-0.750)

Supplementary Table 13. Results of hold-out testing for the ImageNet-pretrained ResNet50 with varied preprocessing approaches. Predictions were generated by an ensemble of the five-fold classification models. Results are reported as the mean and 95% confidence intervals (in brackets) from 10,000 iterations of bootstrapping. The greatest results are shown in **bold**.

Preprocessing Approach	Balanced Accuracy	AUROC	F1 Score
Baseline	69.2% (58.7-79.7%)	0.956 (0.928-0.980)	0.696 (0.582-0.807)
Reinhard Normalisation	75.8% (65.1-86.0%)	0.968 (0.943-0.986)	0.761 (0.647-0.861)
Macenko Normalisation	74.5% (64.3-84.3%)	0.959 (0.933-0.980)	0.756 (0.648-0.857)
Otsu Thresholding	77.2% (66.4-87.6%)	0.963 (0.937-0.985)	0.797 (0.685-0.895)
Otsu + Macenko	**80.5%** (70.4-89.9%)	**0.983** (0.967-0.995)	**0.834** (0.730-0.921)
5× Colour Augmentation	74.9% (63.8-85.6%)	0.966 (0.941-0.986)	0.762 (0.647-0.866)
10× Colour Augmentation	76.1% (65.0-86.6%)	0.962 (0.935-0.983)	0.768 (0.659-0.869)
20× Colour Augmentation	80.0% (69.2-90.0%)	0.973 (0.953-0.989)	0.806 (0.706-0.897)

Supplementary Table 14. Results of external validation on the Transcanadian Study dataset for the ImageNet-pretrained ResNet50 with varied preprocessing approaches. Predictions were generated by an ensemble of the five-fold classification models. Results are reported as the mean and 95% confidence intervals (in brackets) from 10,000 iterations of bootstrapping. The greatest results are shown in **bold**.

Preprocessing Approach	Balanced Accuracy	AUROC	F1 Score
Baseline	52.4% (49.5-55.1%)	0.868 (0.847-0.889)	0.412 (0.380-0.444)
Reinhard Normalisation	51.0% (47.7-54.3%)	0.870 (0.850-0.888)	0.392 (0.350-0.437)
Macenko Normalisation	45.9% (41.8-50.0%)	0.837 (0.814-0.860)	0.407 (0.360-0.455)
Otsu Thresholding	**54.7%** (51.9-57.6%)	**0.883** (0.864-0.901)	**0.440** (0.401-0.482)
Otsu + Macenko	44.4% (40.7-48.3%)	0.840 (0.816-0.862)	0.388 (0.347-0.432)
5× Colour Augmentation	51.7% (48.4-54.8%)	0.867 (0.845-0.887)	0.401 (0.363-0.441)
10× Colour Augmentation	51.1% (47.8-54.2%)	0.877 (0.856-0.897)	0.404 (0.367-0.443)
20× Colour Augmentation	51.4% (48.4-54.4%)	0.874 (0.853-0.893)	0.391 (0.352-0.433)

Supplementary Table 15. Results of external validation on the OCEAN Challenge dataset for the ImageNet-pretrained ResNet50 with varied preprocessing approaches. Predictions were generated by an ensemble of the five-fold classification models. Results are reported as the mean and 95% confidence intervals (in brackets) from 10,000 iterations of bootstrapping. The greatest results are shown in **bold**.

The Reinhard normalisation procedure gave the poorest cross-validation performance. As shown in Supplementary Figure 3, this method was particularly affected by the presence of artifacts in training slides as the normalisation would make these areas appear similar to tissue, making it harder for the attention mechanism to effectively discard these patches. The impact was smaller on Macenko normalisation, which often avoided applying stain colouring to plain background regions. Colour augmentations were unaffected as they could not introduce staining to non-tissue patches (see Supplementary Figure 3).

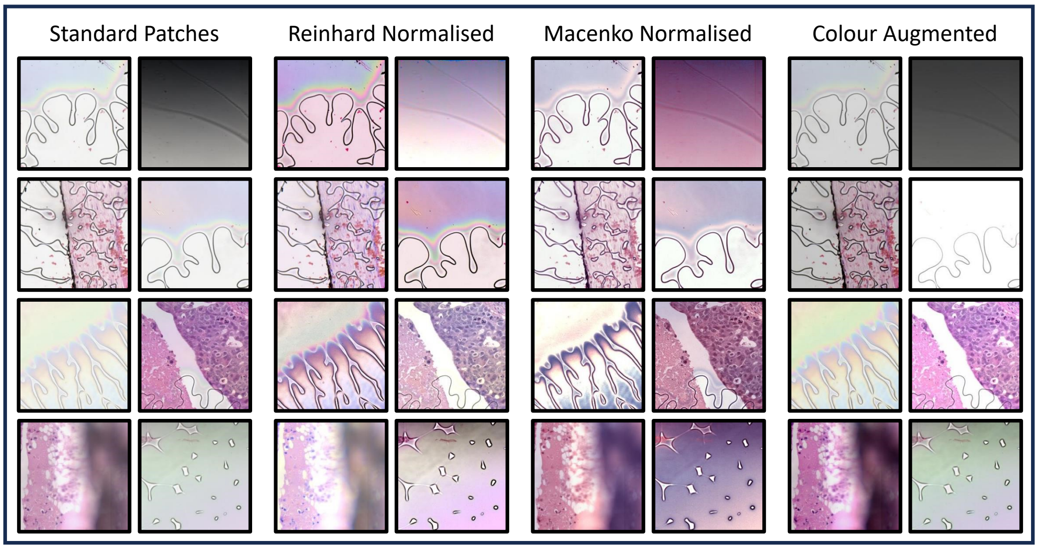

Supplementary Figure 3. Tissue normalisation and augmentation artifacts. Illustrated using 256x256 pixel patches containing artifacts from the same slide at 10× magnification shown in Supplementary Figure 2. The normalisation procedures erroneously apply staining to many non-tissue regions, where the colour augmentations are much less affected. This can be seen most clearly in patches where no tissue is present, such as the bottom-right patch in each group.

The Otsu procedure was found to remove some unstained tissue and artifacts, while also missing some small areas of stained tissue which may have contained diagnostically relevant information (Supplementary Figure 4). It was unclear from the results whether this was beneficial or harmful overall, with Otsu thresholding giving inconsistent performance when compared to the baseline.

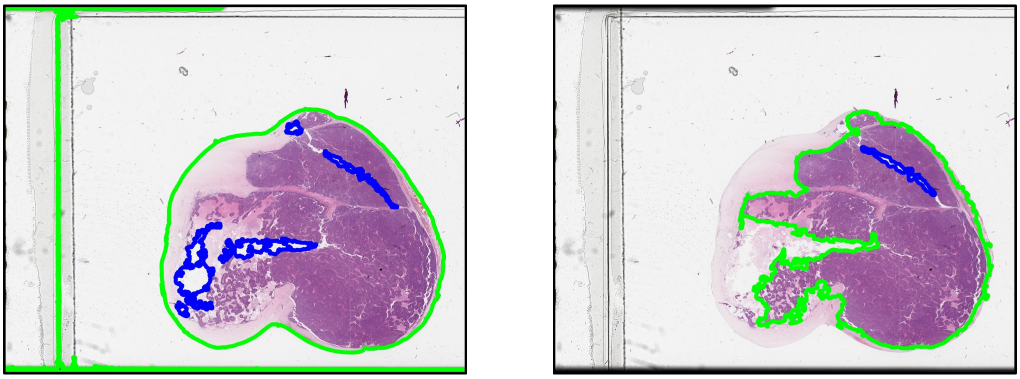

(a) Default saturation thresholding [1]. **(b)** Otsu saturation thresholding [2].

Supplementary Figure 4. Tissue segmentation. Examples of two saturation thresholding tissue segmentation approaches, with green outlines indicating tissue and blue outlines indicating holes within the tissue regions. This example contains a coverslip edge which is incorrectly identified as foreground by the default approach, and a small amount of stained tissue which is excluded by Otsu thresholding.

E RESULTS OF HYPOTHESIS TESTING

Model	Cross-Validation p-values Balanced Accuracy	Cross-Validation p-values AUROC	Cross-Validation p-values F1 Score	Hold-out Testing p-values Balanced Accuracy	Hold-out Testing p-values AUROC	Hold-out Testing p-values F1 Score
RN18	0.736	0.557	0.736	0.601	**0.028**	0.317
ViT-L	**0.033**	0.824	0.074	**0.003**	0.051	**0.002**
RN18-Histo	0.365	0.964	0.613	0.870	0.192	0.967
Lunit	**0.019**	0.183	**0.009**	**0.010**	**0.035**	**0.008**
RN50-Histo	0.232	0.072	0.152	0.214	**0.009**	0.189
CTransPath	**0.009**	0.082	**0.007**	**0.003**	**0.012**	**0.003**
Hibou-B	**0.019**	**0.029**	**0.012**	**0.003**	**0.006**	**0.003**
Phikon	**0.009**	0.149	**0.007**	**0.003**	**0.012**	**0.003**
Kaiko-B8	**0.013**	0.063	**0.010**	**0.003**	**0.011**	**0.002**
GPFM	**0.007**	0.063	**0.007**	**0.003**	**0.006**	**0.003**
UNI	**0.015**	**0.020**	**0.009**	**0.003**	**0.006**	**0.002**
Hibou-L	**0.007**	0.072	**0.007**	**0.003**	**0.009**	**0.003**
Virchow	**0.011**	**0.020**	**0.006**	**0.003**	**0.006**	**0.003**
Virchow2-CLS	**0.011**	0.063	**0.009**	**0.002**	**0.006**	**0.002**
H-Optimus-0	**0.005**	**0.020**	**0.001**	**0.003**	**0.006**	**0.002**
Prov-GigaPath	**0.013**	0.063	**0.007**	**0.008**	**0.006**	**0.008**

Model	Transcanadian Study p-values Balanced Accuracy	Transcanadian Study p-values AUROC	Transcanadian Study p-values F1 Score	OCEAN Challenge p-values Balanced Accuracy	OCEAN Challenge p-values AUROC	OCEAN Challenge p-values F1 Score
RN18	0.446	0.773	0.399	0.237	**0.034**	0.541
ViT-L	**0.021**	0.090	**0.022**	0.170	0.265	**0.019**
RN18-Histo	0.490	0.211	0.403	0.235	0.987	**0.002**
Lunit	**0.003**	**0.011**	**0.006**	**0.002**	**0.004**	**0.001**
RN50-Histo	**0.015**	**0.011**	**0.018**	**0.018**	**0.004**	**0.003**
CTransPath	**0.007**	**0.023**	**0.018**	**0.002**	**0.005**	**0.001**
Hibou-B	**0.008**	**0.024**	**0.007**	0.107	**0.009**	**0.019**
Phikon	**0.002**	**0.011**	**<0.001**	**0.001**	**0.013**	**0.001**
Kaiko-B8	**0.007**	**0.011**	**0.022**	**0.004**	**0.006**	**0.003**
GPFM	**0.003**	**0.011**	**0.006**	**0.001**	**0.004**	**0.001**
UNI	**0.006**	**0.011**	**0.015**	**0.001**	**0.004**	**0.001**
Hibou-L	**0.003**	**0.013**	**0.003**	**0.002**	**0.005**	**0.002**
Virchow	**0.006**	**0.018**	**0.015**	**0.002**	**0.004**	**0.001**
Virchow2-CLS	**0.007**	**0.011**	**0.015**	**0.001**	**0.004**	**0.001**
H-Optimus-0	**0.003**	**0.011**	**0.004**	**0.002**	**0.004**	**0.001**
Prov-GigaPath	**0.019**	**0.017**	**0.035**	**0.005**	**0.004**	**0.002**

Supplementary Table 16. Baseline model comparisons. Resulting p-values from paired t-tests comparing the subtype classification results with each feature extractor to the ImageNet-pretrained ResNet50 baseline. False discovery rate adjustments were applied to account for multiple testing [13]. Values below 0.05 are indicated in **bold**.

Model	Cross-Validation p-values Balanced Accuracy	Cross-Validation p-values AUROC	Cross-Validation p-values F1 Score	Hold-out p-values Balanced Accuracy	Hold-out p-values AUROC	Hold-out p-values F1 Score
RN50	0.617	0.264	0.133	0.171	0.252	0.133
RN18	0.967	0.259	0.170	0.326	0.252	0.170
ViT-L	**0.012**	**0.005**	**0.010**	**0.006**	0.095	**0.010**
RN18-Histo	**0.002**	0.095	0.145	0.086	0.671	0.145
Lunit	0.555	**0.011**	0.168	0.054	0.124	0.074
RN50-Histo	0.864	0.630	0.902	0.912	0.100	0.895
CTransPath	0.144	0.987	**0.042**	**0.030**	0.099	**0.042**
Hibou-B	0.159	0.069	**0.009**	**0.008**	0.207	**0.009**
Phikon	0.709	0.280	0.741	0.619	0.114	0.741
Kaiko-B8	**0.039**	0.089	0.124	0.099	0.063	0.124
GPFM	0.500	**0.029**	0.236	0.262	0.055	0.236
UNI	**0.003**	**0.015**	**0.021**	**0.033**	0.614	**0.021**
Hibou-L	0.104	0.050	0.070	**0.019**	0.193	**0.016**
Virchow	**0.039**	0.059	0.104	0.069	0.076	0.104
Virchow2-CLS	0.194	**0.035**	0.095	0.083	0.108	0.095
H-Optimus-0	0.111	0.069	0.133	0.119	0.089	0.133
Prov-GigaPath	0.412	0.297	0.215	0.194	**0.035**	0.215

Model	Transcanadian Study p-values Balanced Accuracy	Transcanadian Study p-values AUROC	Transcanadian Study p-values F1 Score	OCEAN Challenge p-values Balanced Accuracy	OCEAN Challenge p-values AUROC	OCEAN Challenge p-values F1 Score
RN50	0.190	0.178	0.219	0.303	0.098	0.716
RN18	0.240	0.217	0.106	0.339	0.056	0.279
ViT-L	**0.014**	0.109	**0.014**	**0.001**	**0.006**	**0.021**
RN18-Histo	0.578	0.774	0.973	0.212	0.182	0.620
Lunit	0.099	0.774	0.099	0.104	**0.049**	0.056
RN50-Histo	0.601	0.135	0.479	0.818	**0.023**	0.605
CTransPath	0.853	0.341	0.998	0.790	0.630	0.562
Hibou-B	0.300	0.076	0.286	0.700	0.172	0.590
Phikon	0.740	0.085	0.306	0.119	0.467	0.189
Kaiko-B8	0.213	0.125	0.342	0.102	**0.014**	**0.028**
GPFM	0.386	0.120	0.405	0.861	0.080	0.176
UNI	0.370	0.085	0.959	**0.002**	**0.008**	**0.014**
Hibou-L	0.087	**0.040**	**0.003**	0.142	**0.004**	0.196
Virchow	0.478	0.379	0.460	**0.049**	**0.012**	**0.057**
Virchow2-CLS	0.057	0.871	0.057	**0.017**	0.066	**0.035**
H-Optimus-0	0.167	0.751	0.192	0.866	0.054	0.168
Prov-GigaPath	0.274	0.060	0.209	0.258	0.124	0.416

Supplementary Table 17. Hyperparameter tuning comparisons. Resulting p-values from paired t-tests comparing the subtype classification results for each feature extractor with and without hyperparameter tuning applied to the ABMIL classifier. Values below 0.05 are indicated in **bold**.

	Model 1	Model 2	Bal. Acc. p-value	AUROC p-value	F1 Score p-value
Hold-out Testing	CTransPath	**H-Optimus-0**	0.027	0.037	0.027
	CTransPath	**Hibou-B**	0.013	0.033	0.017
	**CTransPath**	RN18-Histo	0.036	0.013	0.029
	CTransPath	**Virchow2-CLS**	0.013	0.032	0.017
	**GPFM**	RN18-Histo	0.027	0.013	0.023
	**H-Optimus-0**	Kaiko-B8	0.016	0.043	0.017
	**H-Optimus-0**	Lunit	0.026	0.022	0.025
	**H-Optimus-0**	Phikon	0.013	0.017	0.015
	**H-Optimus-0**	RN18-Histo	0.013	0.013	0.015
	**Hibou-B**	Lunit	0.020	0.016	0.020
	**Hibou-B**	Phikon	0.020	0.013	0.025
	**Hibou-B**	RN18-Histo	0.024	0.013	0.020
	**Hibou-L**	Lunit	0.028	0.013	0.035
	**Hibou-L**	Phikon	0.029	0.041	0.039
	**Hibou-L**	RN18-Histo	0.024	0.016	0.020
	**Kaiko-B8**	RN18-Histo	0.021	0.013	0.019
	Kaiko-B8	**Virchow2-CLS**	0.015	0.041	0.017
	Lunit	**UNI**	0.013	0.031	0.015
	Lunit	**Virchow2-CLS**	0.016	0.026	0.017
	Phikon	**UNI**	0.016	0.016	0.017
	Phikon	**Virchow**	0.027	0.016	0.025
	Phikon	**Virchow2-CLS**	0.004	0.016	0.005
	**Prov-GigaPath**	RN18-Histo	0.024	0.013	0.020
	Prov-GigaPath	**Virchow2-CLS**	0.027	0.043	0.025
	RN18-Histo	**UNI**	0.024	0.013	0.020
	RN18-Histo	**Virchow**	0.020	0.013	0.017
	RN18-Histo	**Virchow2-CLS**	0.016	0.013	0.017
Transcanadian Study	CTransPath	**H-Optimus-0**	0.014	0.007	0.003
OCEAN Challenge	**CTransPath**	RN18-Histo	0.006	0.025	0.034
	CTransPath	**UNI**	0.013	0.023	0.034
	**GPFM**	RN18-Histo	0.003	0.021	0.034
	**H-Optimus-0**	RN18-Histo	0.013	0.023	0.042
	Kaiko-B8	**Virchow2-CLS**	0.014	0.023	0.049
	**Lunit**	RN18-Histo	0.007	0.022	0.002
	**Prov-GigaPath**	RN18-Histo	0.012	0.021	0.034
	RN18-Histo	**UNI**	0.003	0.023	0.034
	RN18-Histo	**Virchow**	0.003	0.023	0.024
	RN18-Histo	**Virchow2-CLS**	0.001	0.021	0.034

Supplementary Table 18. Significant pairwise comparisons. Adjusted p-values from pairwise t-tests in which there was a significant difference in all three metrics (this did not occur for any pair of models in cross-validation). The model in **bold** gave the greater balanced accuracy from each pair.

F SUPPLEMENTARY ATTENTION HEATMAPS

Two pathologists (KA and NMO) qualitatively compared the UNI and ImageNet-pretrained attention heatmaps for ten class-balanced example WSIs from the internal hold-out test set. These WSIs (shown in Figure 9, and Supplementary Figures 5 and 6) were selected from those in which a different classification had been determined by each model (specifically using the first-fold model of the five-model ensemble). Out of 39 total disagreements, the UNI-based model gave the correct classification in 26 cases, the ResNet50-based model in 3 cases, and neither was correct in 10 cases. The pathologists were only provided the heatmaps, and were blinded to the models used and the predictions made.

The heatmaps were determined to be similar between models, with both giving high attention to tumour regions and low attention to most stroma regions. Where differences occurred, the ResNet50-based model would typically give high attention to a larger tissue area, often including relevant stromal features (e.g. necrosis and psammoma bodies), but sometimes also including irrelevant stroma. When considering whether heatmaps had focused on diagnostically relevant regions, the pathologists expressed a preference for the UNI-based heatmap in four cases and the ResNet50-based heatmap in three cases, with no preference expressed for the remaining three cases due to their overwhelming similarity. In eight of the selected cases, the UNI model had correctly determined the classification, including all three cases in which the pathologists had preferred the ResNet50-based heatmap. In these cases, the UNI model did not appear to give sufficient attention to all relevant tissue, though it still determined the correct classification. Thus, there was some level of divergence between the pathologists' interpretations and the model heatmaps.

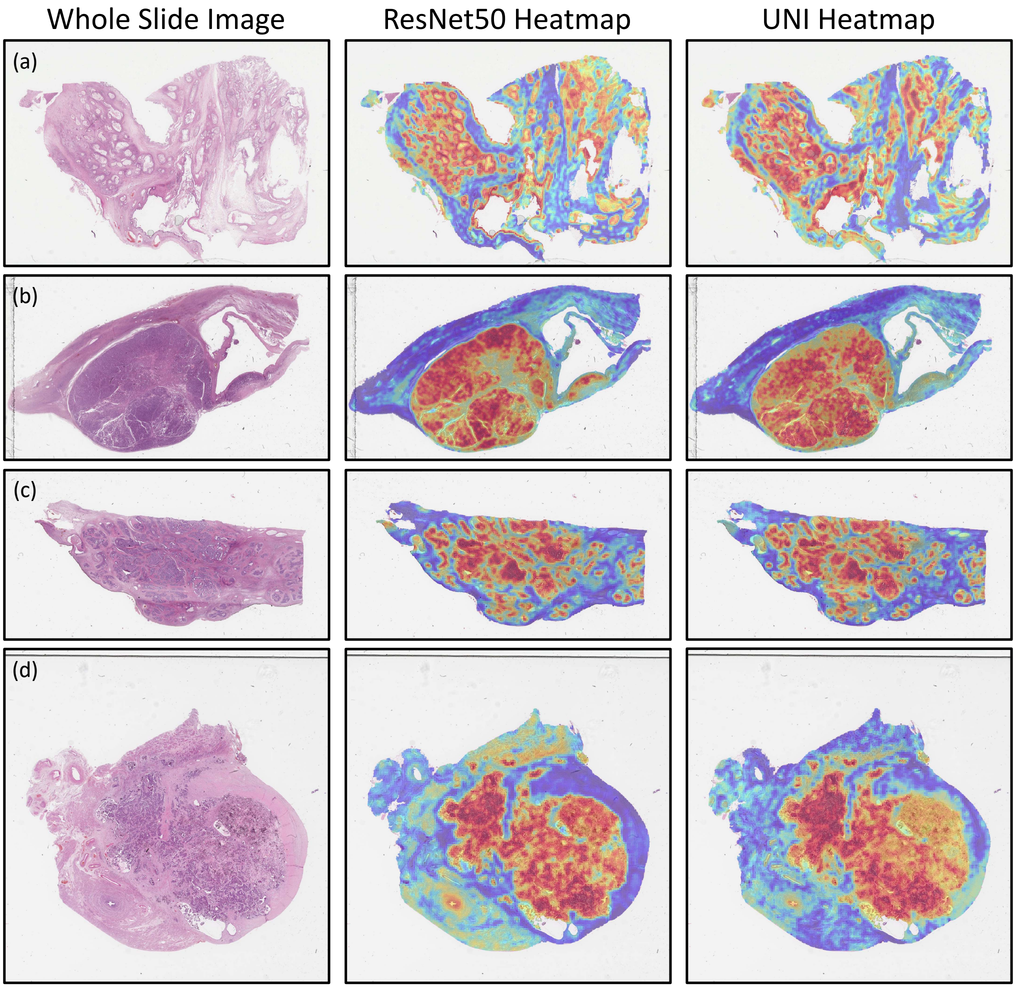

Supplementary Figure 5. Additional heatmaps part 1. Attention heatmaps from the ABMIL classifier using ImageNet-pretrained ResNet50 and UNI foundation model features, where the classification differed between the two models. (a) Ground truth: MC, ResNet50: CCC, UNI: MC. (b) Ground truth: CCC, ResNet50: HGSC, UNI: CCC. (c) Ground truth: EC, ResNet50: HGSC, UNI: EC. (d) Ground truth: LGSC, ResNet50: HGSC, UNI: LGSC.

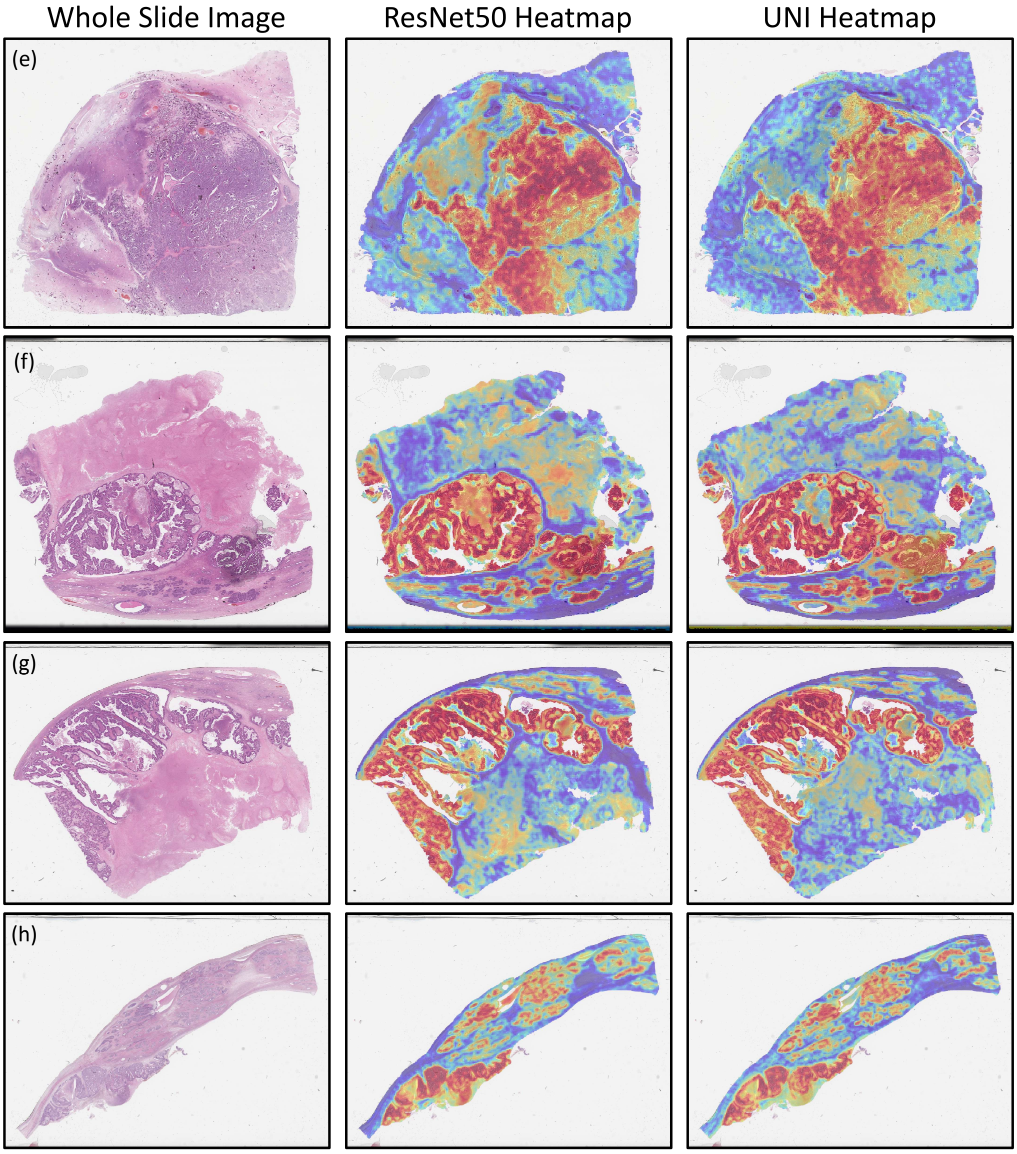

Supplementary Figure 6. Additional heatmaps part 2. Attention heatmaps from the ABMIL classifier using ImageNet-pretrained ResNet50 and UNI foundation model features, where the classification differed between the two models. (e) Ground truth: LGSC, ResNet50: HGSC, UNI: LGSC. (f) Ground truth: HGSC, ResNet50: HGSC, UNI: EC. (g) Ground truth: HGSC, ResNet50: HGSC, UNI: EC. (h) Ground truth: EC, ResNet50: HGSC, UNI: EC.

G TRIPOD+AI REPORTING CHECKLIST

TRIPOD+AI

Version: 11-January-2024

Section/Topic	Item	Development / evaluation[1]	Checklist item	Reported on page
**TITLE**				
Title	1	D;E	Identify the study as developing or evaluating the performance of a multivariable prediction model, the target population, and the outcome to be predicted	1
**ABSTRACT**				
Abstract	2	D;E	See TRIPOD+AI for Abstracts checklist	1
**INTRODUCTION**				
Background	3a	D;E	Explain the healthcare context (including whether diagnostic or prognostic) and rationale for developing or evaluating the prediction model, including references to existing models	2-3
	3b	D;E	Describe the target population and the intended purpose of the prediction model in the context of the care pathway, including its intended users (e.g., healthcare professionals, patients, public)	2-3
	3c	D;E	Describe any known health inequalities between sociodemographic groups	3
Objectives	4	D;E	Specify the study objectives, including whether the study describes the development or validation of a prediction model (or both)	3
**METHODS**				
Data	5a	D;E	Describe the sources of data separately for the development and evaluation datasets (e.g., randomised trial, cohort, routine care or registry data), the rationale for using these data, and representativeness of the data	3-4
	5b	D;E	Specify the dates of the collected participant data, including start and end of participant accrual; and, if applicable, end of follow-up	4
Participants	6a	D;E	Specify key elements of the study setting (e.g., primary care, secondary care, general population) including the number and location of centres	4
	6b	D;E	Describe the eligibility criteria for study participants	4
	6c	D;E	Give details of any treatments received, and how they were handled during model development or evaluation, if relevant	4
Data preparation	7	D;E	Describe any data pre-processing and quality checking, including whether this was similar across relevant sociodemographic groups	4-5
Outcome	8a	D;E	Clearly define the outcome that is being predicted and the time horizon, including how and when assessed, the rationale for choosing this outcome, and whether the method of outcome assessment is consistent across sociodemographic groups	4-5
	8b	D;E	If outcome assessment requires subjective interpretation, describe the qualifications and demographic characteristics of the outcome assessors	4-5
	8c	D;E	Report any actions to blind assessment of the outcome to be predicted	N/A
Predictors	9a	D	Describe the choice of initial predictors (e.g., literature, previous models, all available predictors) and any pre-selection of predictors before model building	4-6
	9b	D;E	Clearly define all predictors, including how and when they were measured (and any actions to blind assessment of predictors for the outcome and other predictors)	4-6
	9c	D;E	If predictor measurement requires subjective interpretation, describe the qualifications and demographic characteristics of the predictor assessors	N/A
Sample size	10	D;E	Explain how the study size was arrived at (separately for development and evaluation), and justify that the study size was sufficient to answer the research question. Include details of any sample size calculation	4-5
Missing data	11	D;E	Describe how missing data were handled. Provide reasons for omitting any data	N/A
Analytical methods	12a	D	Describe how the data were used (e.g., for development and evaluation of model performance) in the analysis, including whether the data were partitioned, considering any sample size requirements	5-8
	12b	D	Depending on the type of model, describe how predictors were handled in the analyses (functional form, rescaling, transformation, or any standardisation).	5-8
	12c	D	Specify the type of model, rationale[2], all model-building steps, including any hyperparameter tuning, and method for internal validation	5-8
	12d	D;E	Describe if and how any heterogeneity in estimates of model parameter values and model performance was handled and quantified across clusters (e.g., hospitals, countries). See TRIPOD-Cluster for additional considerations[3]	N/A
	12e	D;E	Specify all measures and plots used (and their rationale) to evaluate model performance (e.g., discrimination, calibration, clinical utility) and, if relevant, to compare multiple models	8
	12f	E	Describe any model updating (e.g., recalibration) arising from the model evaluation, either overall or for particular sociodemographic groups or settings	N/A
	12g	E	For model evaluation, describe how the model predictions were calculated (e.g., formula, code, object, application programming interface)	8
Class imbalance	13	D;E	If class imbalance methods were used, state why and how this was done, and any subsequent methods to recalibrate the model or the model predictions	8
Fairness	14	D;E	Describe any approaches that were used to address model fairness and their rationale	N/A
Model output	15	D	Specify the output of the prediction model (e.g., probabilities, classification). Provide details and rationale for any classification and how the thresholds were identified	5,8

[1] D=items relevant only to the development of a prediction model; E=items relating solely to the evaluation of a prediction model; D;E=items applicable to both the development and evaluation of a prediction model

[2] Separately for all model building approaches.

[3] TRIPOD-Cluster is a checklist of reporting recommendations for studies developing or validating models that explicitly account for clustering or explore heterogeneity in model performance (eg, at different hospitals or centres). Debray et al, BMJ 2023; 380: e071018 [DOI: 10.1136/bmj-2022-071018]

Page 1 of 2

Supplementary Figure 7. TRIPOD+AI page 1. Transparent Reporting of a multivariable prediction model of Individual Prognosis Or Diagnosis (TRIPOD)+AI Reporting Checklist page 1

TRIPOD+AI

Version: 11-January-2024

Training versus evaluation	16	D;E	Identify any differences between the development and evaluation data in healthcare setting, eligibility criteria, outcome, and predictors	4
Ethical approval	17	D;E	Name the institutional research board or ethics committee that approved the study and describe the participant-informed consent or the ethics committee waiver of informed consent	19
**OPEN SCIENCE**				
Funding	18a	D;E	Give the source of funding and the role of the funders for the present study	19-20
Conflicts of interest	18b	D;E	Declare any conflicts of interest and financial disclosures for all authors	20
Protocol	18c	D;E	Indicate where the study protocol can be accessed or state that a protocol was not prepared	19
Registration	18d	D;E	Provide registration information for the study, including register name and registration number, or state that the study was not registered	19
Data sharing	18e	D;E	Provide details of the availability of the study data	19
Code sharing	18f	D;E	Provide details of the availability of the analytical code[4]	20
**PATIENT & PUBLIC INVOLVEMENT**				
Patient & Public Involvement	19	D;E	Provide details of any patient and public involvement during the design, conduct, reporting, interpretation, or dissemination of the study or state no involvement.	19
**RESULTS**				
Participants	20a	D;E	Describe the flow of participants through the study, including the number of participants with and without the outcome and, if applicable, a summary of the follow-up time. A diagram may be helpful.	5
	20b	D;E	Report the characteristics overall and, where applicable, for each data source or setting, including the key dates, key predictors (including demographics), treatments received, sample size, number of outcome events, follow-up time, and amount of missing data. A table may be helpful. Report any differences across key demographic groups.	4-5
	20c	E	For model evaluation, show a comparison with the development data of the distribution of important predictors (demographics, predictors, and outcome).	4-5
Model development	21	D;E	Specify the number of participants and outcome events in each analysis (e.g., for model development, hyperparameter tuning, model evaluation)	4
Model specification	22	D	Provide details of the full prediction model (e.g., formula, code, object, application programming interface) to allow predictions in new individuals and to enable third-party evaluation and implementation, including any restrictions to access or re-use (e.g., freely available, proprietary)[5]	5-7
Model performance	23a	D;E	Report model performance estimates with confidence intervals, including for any key subgroups (e.g., sociodemographic). Consider plots to aid presentation.	9-14,23-26
	23b	D;E	If examined, report results of any heterogeneity in model performance across clusters. See TRIPOD Cluster for additional details[3].	N/A
Model updating	24	E	Report the results from any model updating, including the updated model and subsequent performance	N/A
**DISCUSSION**				
Interpretation	25	D;E	Give an overall interpretation of the main results, including issues of fairness in the context of the objectives and previous studies	15-19
Limitations	26	D;E	Discuss any limitations of the study (such as a non-representative sample, sample size, overfitting, missing data) and their effects on any biases, statistical uncertainty, and generalizability	15-19
Usability of the model in the context of current care	27a	D	Describe how poor quality or unavailable input data (e.g., predictor values) should be assessed and handled when implementing the prediction model	15-19
	27b	D	Specify whether users will be required to interact in the handling of the input data or use of the model, and what level of expertise is required of users	17-19
	27c	D;E	Discuss any next steps for future research, with a specific view to applicability and generalizability of the model	17-19

From: Collins GS, Moons KGM, Dhiman P, et al. *BMJ* 2024;385:e078378. doi:10.1136/bmj-2023-078378

[4] This relates to the analysis code, for example, any data cleaning, feature engineering, model building, evaluation.
[5] This relates to the code to implement the model to get estimates of risk for a new individual.

Page 2 of 2

Supplementary Figure 8. TRIPOD+AI page 2. Transparent Reporting of a multivariable prediction model of Individual Prognosis Or Diagnosis (TRIPOD)+AI Reporting Checklist page 2

REFERENCES

[1] Lu MY, Williamson DF, Chen TY, et al. Data-efficient and weakly supervised computational pathology on whole-slide images. Nature biomedical engineering. 2021;5(6):555-70.

[2] Otsu N. A threshold selection method from gray-level histograms. Automatica. 1975;11(285-296):23-7.

[3] Janowczyk A, Zuo R, Gilmore H, et al. HistoQC: an open-source quality control tool for digital pathology slides. JCO clinical cancer informatics. 2019;3:1-7.

[4] Shakhawat H, Hossain S, Kabir A, et al. Review of artifact detection methods for automated analysis and diagnosis in digital pathology. In: Artificial Intelligence For Disease Diagnosis And Prognosis In Smart Healthcare. CRC Press; 2023. p. 177-202.

[5] Breen J, Allen K, Zucker K, et al. Efficient subtyping of ovarian cancer histopathology whole slide images using active sampling in multiple instance learning. In: Tomaszewski JE, Ward AD, editors. Medical Imaging 2023: Digital and Computational Pathology. vol. 12471. International Society for Optics and Photonics. SPIE; 2023. p. 1247110.

[6] Breen J, Zucker K, Allen K, et al. Generative Adversarial Networks for Stain Normalisation in Histopathology. In: Applications of Generative AI. Cham: Springer International Publishing; 2024. p. 227-47.

[7] Reinhard E, Adhikhmin M, Gooch B, et al. Color transfer between images. IEEE Computer graphics and applications. 2001;21(5):34-41.

[8] Macenko M, Niethammer M, Marron JS, et al. A method for normalizing histology slides for quantitative analysis. In: 2009 IEEE international symposium on biomedical imaging: from nano to macro. IEEE; 2009. p. 1107-10.

[9] Dooper S, Pinckaers H, Aswolinskiy W, et al. Gigapixel end-to-end training using streaming and attention. Medical Image Analysis. 2023;88:102881.

[10] Shao Z, Dai L, Wang Y, et al. Augdiff: Diffusion based feature augmentation for multiple instance learning in whole slide image. arXiv preprint arXiv:230306371. 2023.

[11] Zaffar I, Jaume G, Rajpoot N, et al. Embedding space augmentation for weakly supervised learning in whole-slide images. In: 2023 IEEE 20th International Symposium on Biomedical Imaging (ISBI). IEEE; 2023. p. 1-4.

[12] Wang Y, Farnell D, Farahani H, et al. Classification of epithelial ovarian carcinoma whole-slide pathology images using deep transfer learning. arXiv preprint arXiv:200510957. 2020.

[13] Benjamini Y, Hochberg Y. Controlling the false discovery rate: a practical and powerful approach to multiple testing. Journal of the Royal statistical society: series B (Methodological). 1995;57(1):289-300.
